# BMP10 accelerated spinal astrocytic activation in neuropathic pain via ALK2/smad1/5/8 signaling

**DOI:** 10.3389/fphar.2024.1426121

**Published:** 2024-08-12

**Authors:** Jiang Bian, Min Luo, Yunyun Tian, Xuejuan Zhang, Bangjian Zhang, Li Yin, Yuehui Zhang

**Affiliations:** ^1^ Department of Anesthesiology, Panzhihua Central Hospital, Panzhihua, Sichuan, China; ^2^ School of Clinical Medicine, Dali University, Dali, Yunnan, China; ^3^ The Third Affiliated Hospital of Zunyi Medical University, The First People’s Hospital of Zunyi, Zunyi, Guizhou, China; ^4^ Scientific Research and Discipline Construction Office, Panzhihua Central Hospital, Panzhihua, Sichuan, China; ^5^ Department of Neurology, Panzhihua Central Hospital, Panzhihua, Sichuan, China

**Keywords:** bone morphogenetic protein 10, activin receptor-like receptors 2, astrocytic activation, neuropathic pain, Smad1/5/8

## Abstract

**Background:**

Astrocytic activation in the spinal dorsal horn contributes to the central sensitization of neuropathic pain. Bone morphogenetic protein (BMP) 10, one of the BMPs highly expressed in the central nervous system, has been demonstrated to have an accelerated effect on astrocytic activation. This study aimed to investigate the functional effects of BMP10 on the activation of astrocytes in the spinal dorsal horn of animal model of neuropathic pain and to explore potential mechanisms involved in this process.

**Methods:**

A neuropathic pain mice model was established using the spared nerve injury (SNI). Western blot analysis was performed to detect the expressional levels of BMP10, activin receptor-like receptor 2 (ALK2), Smad1/5/8, phosphorylated Smad1/5/8, and glial fibrillary acidic protein (GFAP). Immunofluorescence staining was used to detect BMP10, ALK2, and GFAP distribution and expression. The behavioral changes in mice were evaluated using paw withdrawal threshold (PWT), thermal withdrawal latency (TWL), and open field test (OFT). The BMP10 siRNA, Smad1 siRNA, BMP10 peptide, and ALK2-IN-2 (ALK2 inhibitor) were intrathecally administrated to mice. A model of lipopolysaccharide (LPS)-stimulated astrocytes was established to investigate the effect of Smad1. The transfection efficiency of siRNAs was detected by western blot and qRT-PCR analysis.

**Results:**

BMP10 levels were increased in the L4-6 ipsilateral spinal dorsal horn of SNI mice and particularly elevated in astrocytes. Consistently, GFAP and phosphorylated Smad1/5/8 were upregulated in the L4-6 ipsilateral spinal dorsal horn after SNI, indicating the activation of astrocytes and Smad1/5/8 signaling. An intrathecal injection of BMP10 siRNA abrogated pain hypersensitivity and astrocytic activation in SNI mice. In addition, intrathecal administration of BMP10 peptide evoked pain hypersensitivity and astrocytic activation in normal mice, and this action was reversed by inhibiting the ALK2. Furthermore, targeting Smad1 in vitro with the help of siRNA inhibited the activation of astrocytes induced by LPS. Finally, targeting Smad1 abrogated BMP10-induced hypersensitivity and activation of astrocytes.

**Conclusion:**

These findings indicate that the BMP10/ALK2/Smad1/5/8 axis plays a key role in pain hypersensitivity after peripheral nerve injury, which indicates its stimulative ability toward astrocytes.

## Graphical Abstract

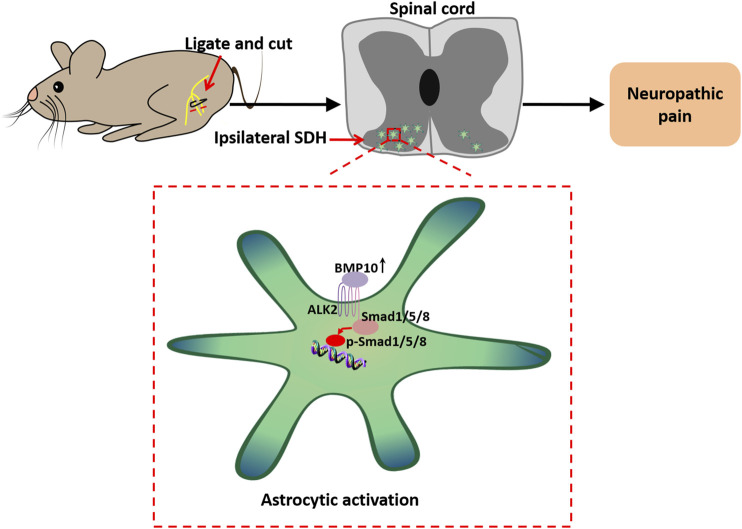

## 1 Introduction

Neuropathic pain refers to pain caused by an injury or disease at the peripheral or central level of the somatosensory nervous system (Bouhassira, 2019). Typical symptoms of neuropathic pain are spontaneous pain, hyperalgesia, and allodynia. An epidemiological study reported that neuropathic pain has a prevalence of up to 10% in the general population and exerts a heavy burden on activities of daily living and working, leading to poor physical and mental qualities of life (Scholz et al., 2019). However, because the potential mechanisms of neuropathic pain remain largely unknown, the diagnostic and therapeutic progress in neuropathic pain remains enormously challenging. Recent research has yielded a deeper understanding of the pathogenesis of neuropathic pain. Neural plasticity, which involves the functional to structural changes in the peripheral nervous system (peripheral sensitization), primarily in the dorsal root ganglion, and in the dorsal part of the spinal cord and supra‐spinal area (central sensitization), is pivotal to the occurrence and maintenance of neuropathic pain (Finnerup et al., 2021). If peripheral sensitization increases pain sensitivity, central sensitization eventually causes neuropathic pain by amplifying pain and reducing the nociceptive threshold (Kuner, 2010). Therefore, the current research on neuropathic pain emphasizes more on the central sensitization mechanisms. Most often, the glial cells in the spinal dorsal horn, including microglia and astrocytes, are polarized by substances such as ATPs or chemokines released by central terminals of the injured afferents (Gao and Ji, 2010). The activated glial cells subsequently facilitate the excitability of the nociceptive neurons in the spinal dorsal horn by releasing abundant inflammatory mediators (Zhang et al., 2023). Consequently, the aberrant excitation of nociceptive neurons leads to the persistent sensitization of nociceptive transmission in the spinal dorsal horn (Descalzi et al., 2015).

Bone morphogenetic proteins (BMPs), members of the transforming growth factor-β (TGF-β) family, were originally recognized as bone inducers (Katagiri et al., 2018). Recently, BMPs have been found to be involved in biological activities of the nervous system, including neural proliferation (Sabo et al., 2009), differentiation (Manzari-Tavakoli et al., 2022), and apoptosis (Pang et al., 2023), and also the maintenance of adult brain and spinal cord homeostasis (Sandy et al., 2023; Okur et al., 2024). BMPs initiate intracellular signaling through a tetrameric receptor kinase complex consisting of BMP type I receptors (BMPR1A, BMPR1B, activin A receptor type I, and activin A receptor–like type I) and BMP receptor type II receptors (BMPR2, activin A receptor type IIA, and activin A receptor type IIB) (Antebi et al., 2017). In general, the activated BMP receptors then phosphorylate the small mothers against decapentaplegic (Smad) 1/5/8, which enter into the nucleus to cooperate with Smad4 to modulate the gene expression (Banerjee et al., 2023). Interestingly, BMPs have been found to play a pleiotropic role in regulating nociception (de la Puerta et al., 2019; Yang et al., 2019; Liu et al., 2022). However, the exact mechanisms are still lacking. BMP10 is abundantly expressed in the CNS, partially in neurons and astrocytes. This expressional profile strongly supports the idea that BMP10 might play an important role in the nervous system (Ogawa et al., 2022). Physiologically, the activin receptor-like kinase 1 (ALK1), the specific type I receptor for BMP10, is involved in fetal brain development (Baello et al., 2014). Pathophysiologically, BMP10 was upregulated in the rat model of cortical brain injury and promoted the proliferation of astrocytes (Yan et al., 2012). Regarding pain, ALK2, another specific type I receptor for BMP10, mediated peripheral analgesia by facilitating the hyperpolarization of dorsal root ganglion neurons (Lin et al., 2021; Yu et al., 2023). ALK1 and ALK2 are known to be widely expressed on numerous cells, initiating the intracellular SMAD1/5/8 signaling induced by BMP10 (Mitrofan et al., 2017; Araki et al., 2018; Ruiz et al., 2020). The involvement of SMAD1/5/8 in pain is well established by its regulation of the excitability of sensory neurons and inflammatory response (Yu et al., 2023; Zhao et al., 2024). It is well known that astrocytic activation is critical for maintaining the potentiation of nociceptive synaptic transmission in the spinal dorsal horn via neuronal-glial and glial-glial communication (Jiang et al., 2016; Ji et al., 2019; Mou et al., 2022). Regarding astrocytes, SMAD1/5/8 was demonstrated to be a key modulator of BMP-induced astrocytic activation in several neurological diseases (Shijo et al., 2018; Nakajima et al., 2021; Skauli et al., 2022). Therefore, the current study aims to investigate whether BMP10 and ALK2/Smad1/5/8 pathway modulate spinal astrocytic activation in mice with SNI and explore the underlying mechanisms.

## 2 Materials and methods

### 2.1 Animal

Male C57BL/6J mice (Charlesriver, China) weighing 20–25 g were housed in two per cage under standard laboratory conditions (environmental temperature 22°C–25°C, 12/12 h light/dark cycle, humidity 50%–60%). As reported previously, the pain perception of males is less susceptible and fluctuant than that of females due to the differences in sex hormone levels and the estrous cycle (Archey et al., 2019; Joshi et al., 2024). Hence, male mice were chosen as the subjects of this study. The mice had access to chow and tap water *ad libitum*. All animal studies were approved by the Institutional Animal Care and Use Committee of Southwest Medical University (20220809-005). All experimental procedures were rigorously performed in accordance with the Guide for the Care and Use of Laboratory Animals of the National Institutes of Health.

### 2.2 Animal grouping

**FIGURE 1:**
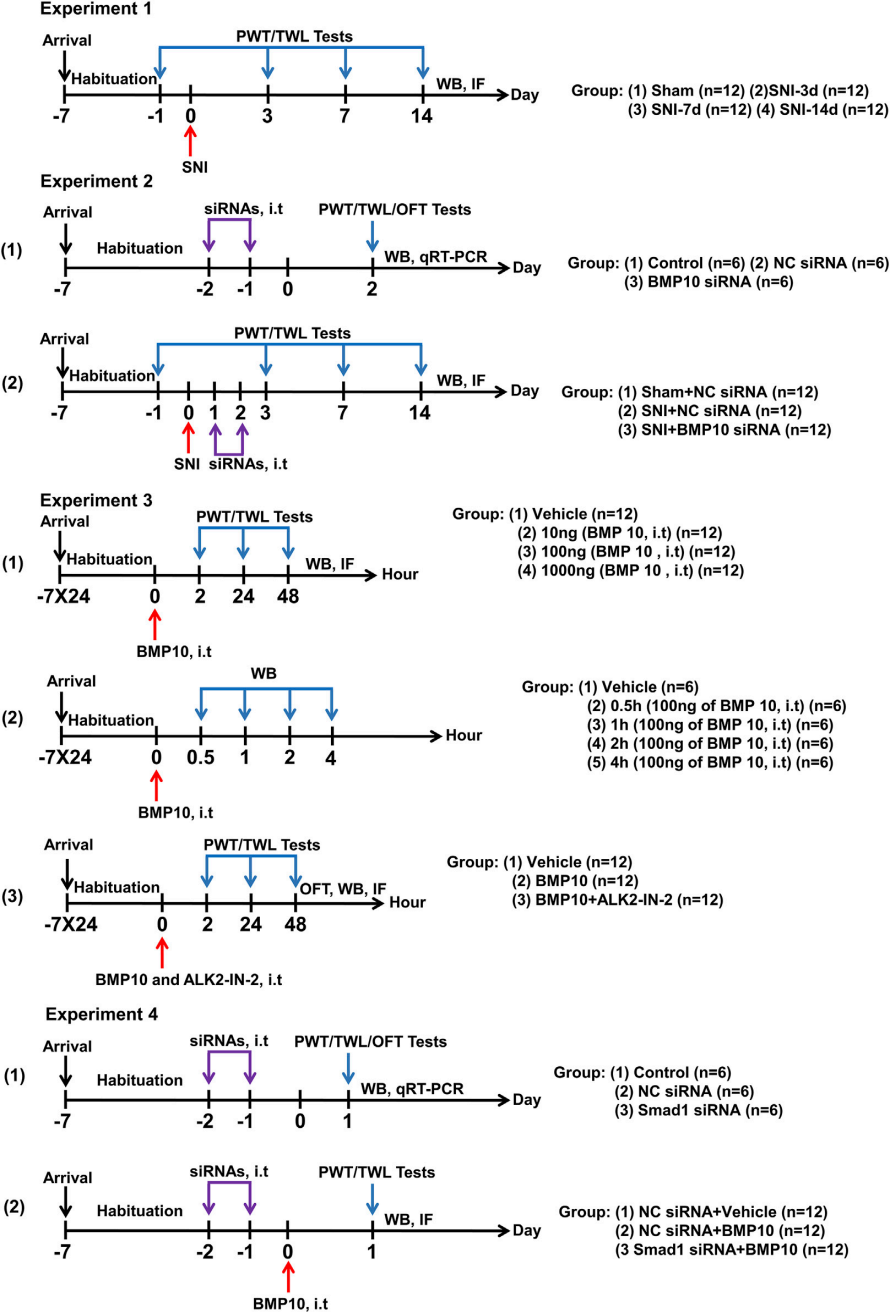
Graphical representation of the experimental design. Experiment 1: Changes in pain sensitization and expressions of BMP10, ALK2, Smad1/5/8, phosphorylated Smad1/5/8, and GFAP after SNI in mice. Experiment 2: The effects of BMP10 siRNA on SNI-induced pain hypersensitivity and astrocytic activation. Experiment 3: The involvements of ALK2 in BMP10-induced pain hypersensitivity and astrocytic activation. Experiment 4: The effects of Smad1 siRNA on BMP10-induced pain hypersensitivity and astrocytic activation.

The animal experiment contained four parts (Figure 1):

#### 2.2.1 Changes in pain sensitization and expressions of BMP10, ALK2, Smad1/5/8, phosphorylated Smad1/5/8, and GFAP after SNI in mice

In all, 48 mice were randomly divided into Sham, SNI-3d, SNI-7d, and SNI-14d groups (n = 12 per group). Paw withdrawal threshold (PWT) and thermal withdrawal latency (TWL) were measured on baseline (Day 1 before SNI surgery), Days 3, 7, and 14 after SNI surgery. Mice were dissected on Days 3, 7, and 14 after SNI surgery for immunofluorescence staining (n = 6 per group) and western blot analysis (n = 6 per group) to investigate the expression levels of BMP10, ALK2, Smad1/5/8, phosphorylated Smad1/5/8 and GFAP.

#### 2.2.2 Intrathecal injection of BMP10 siRNA ameliorated mechanical and thermal hypersensitivity and the activation of astrocytes caused by SNI

(1) To identify the blockade activity of BMP10 siRNA targeting gene expression, 18 mice were randomly allocated to the Control group, NC siRNA group, and BMP10 siRNA group (n = 6 per group). PWT and TWL were measured to evaluate the sensory function. The open field test (OFT) was performed to detect motor function and anxiety-like behavior. Western blot and qRT-PCR analysis were performed to verify the transfection efficiency.
(2) To explore whether BMP10 siRNA ameliorated pain hypersensitivity and astrocytic activation of SNI mice, 36 mice were randomly divided into three groups (n = 12 per group): Sham + NC siRNA group, SNI + NC siRNA group, and SNI + BMP10 siRNA. PWT and TWL were measured on baseline (Day 1 before SNI surgery), Days 3, 7, and 14 after SNI surgery. Western blot was performed to detect the expression levels of BMP 10 and GFAP (n = 6 per group); immunofluorescence staining was performed to detect the expression of GFAP (n = 6 per group).

#### 2.2.3 Blockade of ALK2 abolished the effects of BMP10 on pain hypersensitivity and astrocytic activation in normal mice

(1) To determine whether a single intrathecal injection of BMP10 could induce pain hypersensitivity and activation of astrocytes, 48 mice were randomly divided into four groups (n = 12 per group): Vehicle group, 10 ng of BMP10 group, 100 ng of BMP10 group, and 1,000 ng of BMP10 group. The PWT and TWL were measured at baseline (pre-injection), 2 h, 24 h, and 48 h after BMP10 injection. Western blot was performed to detect the expression level of GFAP in the spinal cord (n = 6 per group), immunofluorescence staining was performed to detect the expression of c-fos, calcitonin gene-related peptide (CGRP), Ionized Calcium Binding Adaptor Molecule 1 (Iba-1) (n = 6 per group).
(2) To identify the activity of exogenous BMP10 in the spinal cord, 30 mice were randomly divided into Vehicle group (n = 6 per group) and BMP10 groups (0.5 h post-injection (n = 6 per group), 1 h post-injection (n = 6 per group), 2 h post-injection (n = 6 per group), and 4 h post-injection (n = 6 per group)). Western blot was performed to detect the BMP10 level in the spinal cord.
(3) To explore whether ALK2 inhibitor could reverse the effect of BMP10, 36 mice were randomly divided into three groups (n = 12 per group): Vehicle group, BMP10 group, BMP10+ALK2-IN-2 group. The PWT and TWL were measured at baseline (pre-injection), 2 h, 24 h, and 48 h after BMP10 injection. Immunofluorescence staining (n = 6 per group) and western blot (n = 6 per group) were performed to detect the GFAP expression level in the spinal cord.

#### 2.2.4 Smad1 siRNA abrogated BMP10 induced-pain hypersensitivity and astrocytic activation

(1) To identify the blockade activity of Smad1 siRNA targeting gene expression, 18 mice were randomly allocated to the Control group, NC siRNA group, and Smad1 siRNA group (n = 6 per group). PWT and TWL were measured to evaluate the sensory function. The OFT was performed to detect motor function and anxiety-like behavior. Western blot and qRT-PCR analysis were performed to verify the transfection efficiency.
(2) To identify whether Samd1/5/8 was involved in BMP10-induced astrocytic activation and pain hypersensitivity, 36 mice were randomly divided into three groups (n = 12 per group): NC siRNA + Vehicle group, NC siRNA + BMP10 group, and Samd1 siRNA + BMP10. PWT and TWL were measured at 24 h after BMP injection. Western blot was performed to detect the p-Samd1/5/8 and GFAP expression levels (n = 6 per group); immunofluorescence analysis was performed to detect the GFAP expression levels (n = 6 per group).

### 2.3 SNI-induced neuropathic pain

It is well accepted that SNI could trigger long-lasting pathological responses, such as robust glial activation and marked neuronal hypersensitivity, in dorsal root ganglion, spinal dorsal horn, and supraspinal areas, contributing to the peripheral and central sensitization of neuropathic pain (Guida et al., 2020). Therefore, the SNI model was performed to mimic the neuropathic pain in mice, and the SNI model was constructed as described in previous literature (Decosterd and Woolf, 2000). In brief, mice were anesthetized by inhalation of 2.0%–4.0% isoflurane, and a transverse incision of approximately 1–2 cm was made near the superior border of the right hind limb. The sciatic nerve trunk near the thigh region was exposed, and its branches, including the common peroneal nerve, tibial nerve, and sural nerve, were isolated. The tibial and common peroneal nerves near the trifurcation were tightly ligated using 5–0 silk sutures. Subsequently, the distal part of the ligation was cut off, and 1–3 mm of the distal nerve end remained. For sham surgery mice, the three branches of the sciatic nerve were gently separated at the same site on the right thigh, while the tibial and common peroneal nerves were not ligated. The incision was stitched layer by layer and disinfected with iodophor. After the operation, all mice recovered on a heated blanket and were monitored for signs of infection.

### 2.4 Behavioral tests

PWT was measured to evaluate the mechanical allodynia. In brief, the mice were placed in elevated chambers (10 cm × 5 cm × 5 cm) with a wire mesh floor for 30 min before PWT testing. A series of Von-Frey filaments (North Coast Medical, CA, United States) from 0.02 to 2 g were incrementally stabbed to the plantar surface of the hind paw with an ascending force to bend the filaments for 3 s slightly. A positive response was determined as a quick withdrawal of the tested hind paw. When a single stimulus force induced more than three positive responses in five tests, it was chosen as the recorded PWT.

TWL to radiant heat stimulus was measured to assess thermal hyperalgesia. Before the behavioral experiment, the mice were placed in elevated chambers (10 cm × 5 cm × 5 cm) with a plexiglass floor for 30 min. Subsequently, a radiant heat source (Yuyan Instruments Company, Shanghai, China) was focused on the center area of the plantar surface of the hind paw three times at 5-min intervals. The duration from the start to the paw withdrawal was determined as the withdrawal latency. The average withdrawal latency of three repeats was considered as the PWT.

Open field test (OFT) was performed to assess the locomotor activity and anxiety-like behavior as previously described (Wu et al., 2022). In brief, a high-resolution video camera measured motor activity in an open field (45 cm × 45 cm × 30 cm). Each mouse was placed in the open field’s center zone and allowed to explore for 5 min freely. After a single test, 75% alcohol was used to clean the entire area of the open field. The captured video was processed using a video tracking system (ANY-maze V6.14, Stoelting, United States). The locomotor function was evaluated by calculating the total distance and average speed.

### 2.5 Intrathecal injection

Drugs were intrathecally administrated into the subarachnoid space of mice following the lumbar puncture method as described in previous literature (Fairbanks, 2003). Following inhalation of 2.0% isoflurane, the mice were fixed in the prone position on a stereotaxic instrument. Further, a 5-μL Hamilton microsyringe with a 20-gauge needle was inserted into subarachnoid space through L5-L6 intervertebral spaces. The sudden tail flick was considered a sign of a successful puncture. After intrathecal injection, the mice with motor dysfunction were excluded from the following experiments.

### 2.6 Drug administration

To examine the effects of BMP10 in mice, mouse BMP10 peptide (6038-BP, Novus Biologicals, CO, United States) was intrathecally administrated in a dose-dependent manner. Subsequently, 10, 100, and 1,000 ng of BMP10 peptide were dissolved in 2 μL normal saline. To ensure peptide bioactivity *in vivo*, the prepared BMP10 peptide solutions were administrated to mice within 15 min. ALK2 selective inhibitor ALK2-IN-2 (100 ng) (HY-112815, MedChemExpress, NJ, United States) was intrathecally administrated with the BMP10 peptide (100 ng) to find the receptor responsible for BMP10’s effects in mice. The dosages of ALK2-IN-2 were determined following the manufacturer’s instructions and as per the preliminary experiments. ALK2-IN-2 was dissolved in 2 μL of 10%DMSO (HY-Y0320, MedChemExpress, NJ, United States), and 10%DMSO was considered the vehicle. Next, 100 ng of BMP10 peptide was added to ALK2-IN-2 and vehicle solution and intrathecally injected within 15 min.

### 2.7 *In vivo* siRNA transfection

For *in vivo* siRNA transfection, siRNA targeting BMP10, Smad1, and negative control (NC) siRNA were obtained from OBIO Technology (Shanghai, China). The sequence of BMP10 siRNA was as follows: sense 5′-CGG​AGC​AAG​AUG​GUA​UUG​ACU​UCA​A-3′ and anti-sense 5′-UUG​AAG​UCA​AUA​CCA​UCU​UGC​UCC​G-3′. The sequence of Smad1 siRNA was as follows: sense 5′-GAG​AAA​GCU​GUG​GAC​GCU​UUG​GUG​A-3′ and anti-sense 5′-UCA​CCA​AAG​CGU​CCA​CAG​CUU​UCU​C-3′. A scrambled sequence of nucleotides was selected as NC siRNA: sense 5′-CGG​GAA​CGG​UAU​UAU​UCA​GUG​ACA​A-3′ and anti-sense 5′-UUG​UCA​CUG​AAU​AAU​ACC​GUU​CCC​G-3′. The BMP10 siRNA, Smad1 siRNA, and NC siRNA were dissolved in a mixture containing RNase-free water (ZeyeTechnology, Shanghai, China), transfection reagent branched polyethyleneimine (4052836, Thermo Fisher Scientific, MA, United States), and 5% glucose to make a concentration of 2 μg/μL. 4 μg of BMP10 siRNA, Smad1 siRNA or NC siRNA was intrathecally administrated for two consecutive days. The transfection efficiency of siRNA was detected by western blot and qRT-PCR analysis.

### 2.8 *In vitro* siRNA transfection

The mouse spinal cord astrocyte cell line (M1830-57, ScienCell Research Laboratory, United States) was grown in Dulbecco’s modified Eagle’s Medium (DMEM) supplemented with 10% FBS at 37°C with 5% CO_2_. The purity of the astrocyte cell line was determined by immunofluorescence staining using GFAP, an astrocytic marker. Then, 60 nM Smad1 siRNA (OBIO technology, Shanghai, China) or NC siRNA (OBIO technology, Shanghai, China) were transfected into cultured cells with lipofectamine^TM^RNAi MAX (Thermo Fisher Scientific, United States) following the manufacturer’s instructions. After 24 h of transfection, the medium containing siRNAs was replaced with a fresh medium. The lipopolysaccharide (LPS) (100 ng/mL, MedChemExpress, United States) was then supplemented into the medium to stimulate the activation of astrocytes, as described in a previous study (Shao et al., 2021). After 12 h of culture, the cells were harvested for further detection.

### 2.9 Immunofluorescence staining

For immunofluorescence staining of spinal cord tissues, the mice were deeply narcotized using 4% isoflurane and then intracardially perfused with 0.01 M phosphate buffer solution (PBS) followed by 4% paraformaldehyde. The spinal cord tissues were quickly separated on ice. Next, the L4-L6 segments were isolated by tracing the sciatic nerve trunk and branches. The spinal cord samples were transferred into 4% paraformaldehyde for 6 h and were immersed into 30% sucrose solution for 12 h at 4°C. The transverse sections (20 μm) of L4-L6 spinal cord samples were placed in a cryostat (CM 1900, Leica, Wetzlar, Germany). The sections were washed three times to eliminate optimal cutting temperature compound and blocked with 10% goat serum and 0.5% Triton X-100 for 30 min at room temperature. Subsequently, the tissues were incubated with the primary antibodies for 12 h at 4°C as follows: mouse anti-GFAP antibody (1:200, ab279289, Abcam), mouse anti-CGRP antibody (1:500, ab81887, Abcam), mouse anti-c-fos antibody (1:1,000, ab208942, Abcam), mouse anti-Iba-1 antibody (1:200, ab283319, Abcam), rat anti-BMP10 antibody (1:200, 654319, Novus Biologicals, CO, United States) and rabbit anti-ALK2 antibody (1:100, PA5-114818, Thermo Fisher Scientific, MA, United States). After rinsing three times in PBS with Tween 20 (PBST), the tissues were incubated with secondary antibodies for 2 h at room temperature as follows: goat anti-mouse IgG H&L (Alexa Fluor^®^ 488) (1:500, ab150113, Abcam), goat anti-rat IgG H&L (Alexa Fluor^®^ 568) (1:500, ab175476, Abcam), goat anti-rabbit IgG H&L (Alexa Fluor^®^ 555) (1:500, ab150170, Abcam). After rinsing three times in PBST, the tissues were stained with 4,6-diamino-2-phenyl indole (DAPI) solution and anti-fluorescent mounting media. For immunofluorescence staining of cultured cells, glass slides were used to collect the astrocyte cells that were transfected with siRNAs and treated with LPS. Subsequently, the cells were fixed with prepared paraformaldehyde for 20 min and permeabilized with 0.5% Triton X-100 for 10 min. The nonspecific cross-reacting antigens were blocked with 5% goat serum and 5% bovine serum albumin for 30 min at room temperature. The cells were incubated with mouse anti-GFAP antibody (1:200, ab279289, Abcam) for 12 h at 4°C. After rinsing three times in PBST, the cells were incubated with the goat anti-mouse IgG H&L (Alexa Fluor^®^ 488) (1:500, ab150113, Abcam) secondary antibody for 2 h at room temperature. After rinsing three times in PBST, the cells were stained with DAPI solution and anti-fluorescent mounting media. The fluorescence images of the spinal cord sections and cells were acquired using a confocal microscope. To quantify the expression of target proteins, the spinal dorsal horn images (magnification, ×200) were analyzed with the regions of interest (ROIs) using the NIH ImageJ software. Every third section (40 μm apart) was averaged per mouse, with six mice in each group. The percentage of positive fluorescence area was calculated as previously described (Cao et al., 2021). In particular, the threshold of images was standardized based on the same parameters to maximize true protein positive signal for calculation. The percentage of target protein was quantified by the total pixel value of target protein/total unfiltered pixel value in the ROI. In addition, the percentage of colocalization area of GFAP with BMP10 in ROI was calculated to represent the expression of BMP10 in astrocytes. The values in three sections (40 μm apart) for each L4-L6 spinal cord sample were averaged per mouse, with six mice in each group. The GFAP-positive cells in the cultured astrocytes were quantified under ×400magnification and were manually counted. GFAP-positive cells in four different ×400magnification fields were averaged per well, with six repeats in each group.

### 2.10 Western blot

Mice were sacrificed after inhalation of 4% isoflurane. The spinal cord was quickly acquired and transferred on dry ice. The approximately 5 mm L4-L6 spinal cord segments or its ipsilateral spinal dorsal horn were separated from the frozen spinal cords and stored at −80°C. The harvested spinal cord tissues and cultured astrocytes were lysed with radioimmunoprecipitation assay buffer and centrifuged (12,000 rpm) at 4°C for 30 min to extract the supernatants. The concentration of total protein was determined by the BCA method (A53225, Thermo Fisher Scientific, MA, United States) according to the manufacturer’s instructions. 30 ug of protein samples were mixed with loading buffer and transferred to a water bath at 95°C for 10 min. After electrophoretic separation, proteins were transferred to polyvinylidene fluoride membranes (FFP36, Beyotime Biotechnology, Shanghai, China). Subsequently, membranes were incubated with 5% bovine serum albumin for 60 min at room temperature to block non-specific antigens. The membranes were incubated with appropriate antibodies, including mouse anti-GFAP antibody (1:1,000, ab279289, Abcam), mouse anti-BMP10 antibody (1:1,000, 462732, Novus Biologicals, CO, United States), rabbit anti-ALK2 antibody (1:100, PA5-114818, Thermo Fisher Scientific, MA, United States), rabbit anti-phospho-Smad1/5/8 (1:1,000, AB3848-I, Sigma Aldrich, Germany), rabbit anti-Smad1/5/8 (1:1,000, NB100-56656, Novus Biologicals, CO, United States) and mouse anti-β-actin (1:1,000, AF2815, Beyotime Biotechnology, Shanghai, China) overnight at 4°C. The membranes were washed three times with Tris-buffered saline with Tween 20 (TBST) and then incubated with horseradish peroxidase-conjugated goat anti-rabbit antibody (1:3,000, 31460, Invitrogen, United States) or horseradish peroxidase-conjugated goat anti-mouse antibody (1:3,000, 31430, Invitrogen, United States) for 2 h at room temperature. The bands were finally illuminated using the ECL kit (P0272FT, Beyotime Biotechnology, Shanghai, China). Immunoblot images were captured using an LI-COR/Odyssey infrared imaging system (Bio-Rad, ChemiDoc XRS+, United States). The blot density of the bands was quantified using ImageJ software. Fold changes of target protein expression were normalized to those of β-actin.

### 2.11 Quantitative real-time (qRT)-PCR

qRT-PCR was performed to validate the transfection efficiency of siRNAs. L4-L6 spinal cord tissues were prepared as described in the western blot method. Total RNA from L4-L6 spinal cords was extracted using RNA isolator total RNA extraction reagent (R401-01, Vazyme Biotechnology, Nanjing, China) following the manufacturer’s instructions. The concentration of RNA in samples was determined by using the NanoDrop 2000 spectrophotometer (Thermo Fisher Scientific, MA, United States). cDNA was generated by reverse transcription of 100 ng of total RNA with PrimeScript™ RT Master Mix (RR036A, Takara, Japan). Following the standard conditions, the qRT-PCR reactions were run on a real-time PCR instrument (Roche, Switzerland). The relative expression levels of target genes were analyzed following the 2^−ΔΔCT^ method normalized to the internal control genes (GAPDH). The primers used were as follows: BMP10:sense: 5′-ATG​GGG​TCT​CTG​GTT​CTG​C-3′; anti-sense: 5′-CAA​TAC​CAT​CTT​GCT​CCG​TGA​A-3′, Smad1: sense: 5′-CCG​AGC​CGG​CGC​TAA​C-3′ anti-sense: 5′-CAT​TCA​TAG​CGG​CTG​GTC​TAG​T-3′, GAPDH: sense: 5′-AGG​TCG​GTG​TGA​ACG​GAT​TTG-3′ anti-sense: 5′-TGT​AGA​CCA​TGT​AGT​TGA​GGT​CA-3′.

### 2.12 CCK-8 assay

The influence of siRNA on the viability of cultured astrocyte cell lines was detected using the CCK-8 kit (C008, Beyotime Biotechnology, Shanghai, China). After transfection with siRNA and treatment with LPS, astrocytes were seeded in 96-well plates (adjusted density: 1 × 10^4^ cells/well). Following the manufacturer’s instructions, cells in each well were incubated with 10 μL of CCK-8 for 1 h. Finally, the viability of cells was calculated by determining the absorbance at 450 nm using a spectrophotometer (Bio-Rad, CA, United States).

### 2.13 Statistical analysis

Data were processed using SPSS 17.0 software (SPSS Inc., Chicago, IL, United States). The normality and equal variance were determined by the Shapiro-Wilk normality test and Brown-Forsythe test. Comparisons of PWT and TWL between multiple groups at different time points were performed by repeated measure ANOVA, followed by Bonferroni’s *post hoc* test. The comparison of Western blot, Immunofluorescence staining, and qRT-PCR data was performed by one-way ANOVA, followed by Bonferroni’s *post hoc* test or independent sample t-test. Nonparametric data were evaluated using the Kruskal–Wallis test, followed by Dunn’s test. The level of statistical significance was set at *P* < 0.05.

## 3 Results

### 3.1 Algesimetric assessments in mice with SNI-induced neuropathic pain

**FIGURE 2:**
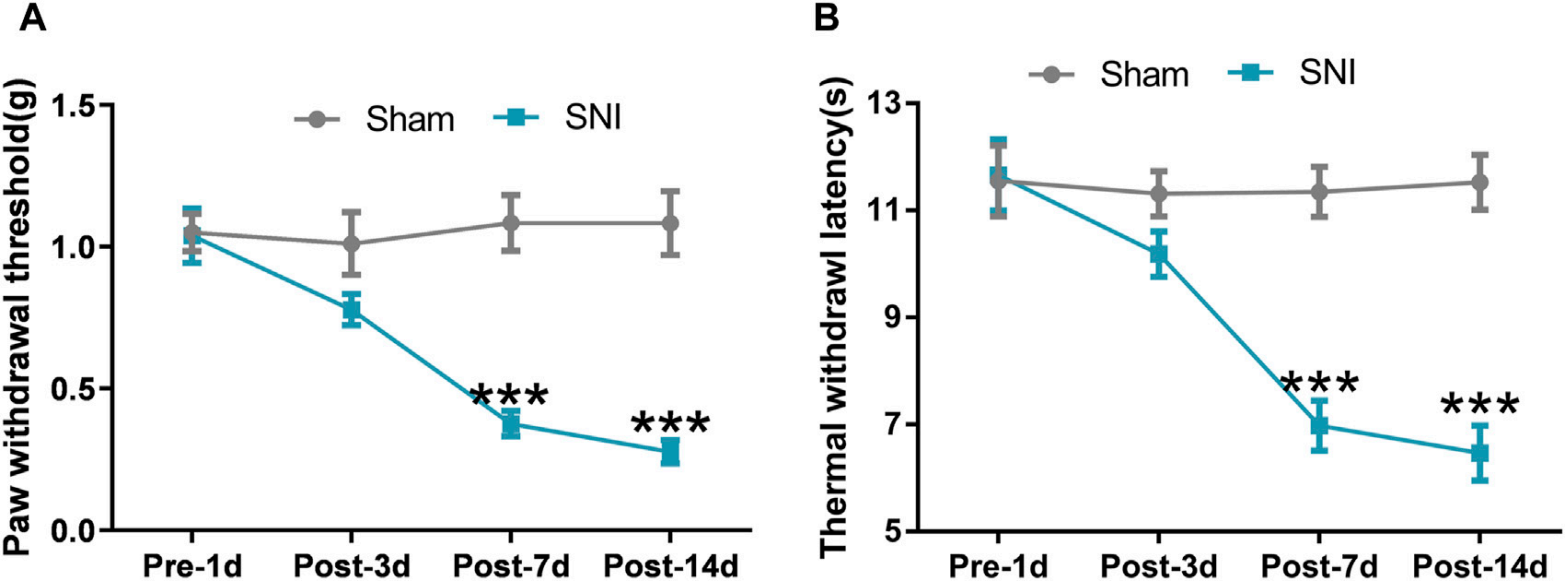
SNI induced neuropathic pain in mice. **(A)** Paw withdrawal threshold (PWT) of mice that underwent SNI or sham surgery was detected on preoperative Day 1 and postoperative Day 3, 7, and 14; **(B)** Thermal withdrawal latency (TWL) of mice that underwent SNI or sham surgery was detected on preoperative Day 1 and postoperative Day 3, 7, and 14. Data are presented as mean and SEM; sham mice versus SNI mice on postoperative Day 7 and 14 *p < 0.05, ***p < 0.001; n = 12.

We performed a unilateral SNI surgery in mice, which could induce obvious and long-lasting mechanical allodynia and thermal hyperalgesia (Guida et al., 2020). SNI-induced neuropathic pain was evaluated through the induction of mechanical allodynia and thermal hyperalgesia of the ipsilateral hind paw with the help of Von-Frey filaments and radiant heat stimulus, respectively. In the Von-Frey filament test, mice with SNI developed apparent mechanical allodynia on postoperative Day 7 compared with mice that underwent sham surgery. Significant mechanical allodynia was maintained until postoperative Day 14, following which repetitive Von-Frey measurement was stopped (Figure 2A) (Repeated measure ANOVA F_1,22_ = 23.267, *P* < 0.0001). Under radiant heat stimulus, mice with SNI exhibited significant thermal hyperalgesia on postoperative Day 7 compared to mice that underwent sham surgery. Thermal hyperalgesia was maintained until postoperative Day 14, following which radiant heat stimulus testing was stopped (Figure 2B) (Repeated measure ANOVA F_1,22_ = 36.432, *P* < 0.0001). The algesimetric assessments indicated that SNI surgery successfully induced classic symptoms of neuropathic pain.

### 3.2 SNI triggered the upregulation of BMP10 and p-Smad1/5/8 and the activation of astrocytes in the ipsilateral spinal dorsal horn

**FIGURE 3:**
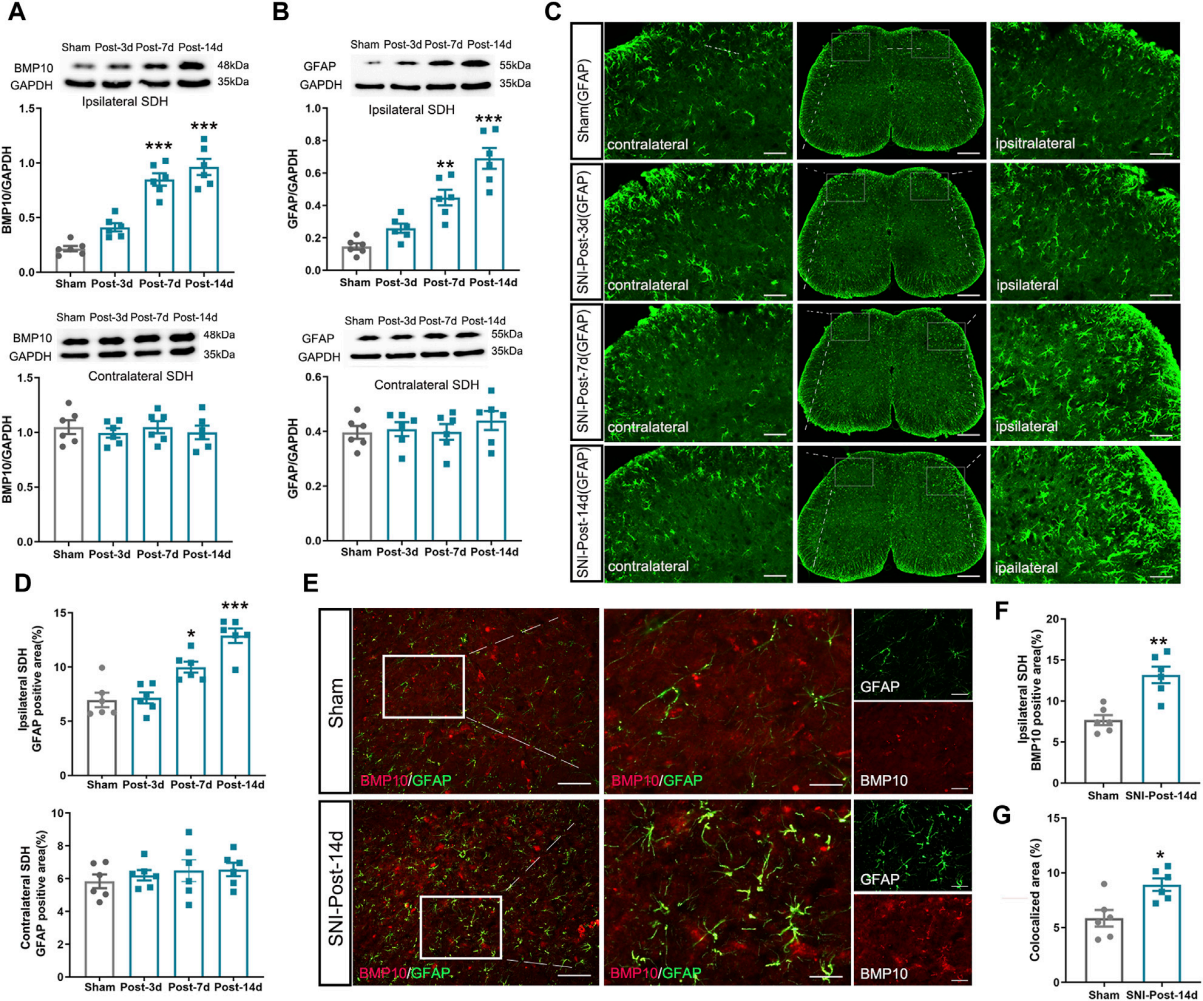
Expression levels of BMP10, GFAP in mouse ipsilateral spinal dorsal horn after SNI. **(A, B)** Western blot analysis showed the expression levels of BMP10 and GFAP in the ipsilateral (above) and contralateral (below) spinal dorsal horn of sham and SNI mice; **(C, D)** Immunofluorescence staining showed the expression of GFAP in the ipsilateral and contralateral spinal dorsal horn of sham and SNI mice (scale bar = 200 μm/50 μm); **(E–G)** Double immunofluorescence staining showed the coexpression of BMP10 (red) with GFAP (green) in the ipsilateral spinal dorsal horn of sham and SNI mice on postoperative Day 14 (scale bar = 200 μm/50 μm). Data are presented as mean and SEM; sham mice versus SNI mice on postoperative Days 7 and 14, ***p < 0.001 (n = 6).

To examine the potential role of BMP10 in neuropathic pain, the expression levels of BMP10 in the spinal dorsal horn were detected on postoperative Day 3, 7, and 14. We found that the protein level of BMP10 in the ipsilateral spinal dorsal horn of SNI mice was increased on postoperative Day 7 and 14 in comparison with the sham mice (Figure 3A) (Ipsilateral spinal dorsal horn: One-way ANOVA F_3,20_ = 41.18, *P* < 0.0001). However, the expression of BPM10 in the contralateral spinal dorsal horn remained unchanged after SNI (Contralateral spinal dorsal horn: One-way ANOVA F_3,20_ = 0.27, *P* = 0.844). As the most abundant cells in the CNS, the activation of astrocytes in the spinal dorsal horn contributes to the central sensitization of neuropathic pain via the glia-neuron crosstalk (Ji et al., 2019). Therefore, we determined the expression of GFAP (astrocytic marker) to investigate the activity of astrocytes in SNI-induced neuropathic pain. Notably, results of western blot and immunofluorescence staining showed that the GFAP protein expression was upregulated in the ipsilateral spinal dorsal horn of SNI mice compared with sham mice on postoperative Day 7 and 14 (Figures 3B–D) (Ipsilateral spinal dorsal horn: GFAP: One-way ANOVA F_3,20_ = 29.40, *P* < 0.0001; GFAP positive area: One-way ANOVA F_3,20_ = 22.19, *P* < 0.0001), indicating the activation of astrocytes after nerve injury. As expected, SNI did not induce the upregulation of GFAP in the contralateral spinal dorsal horn (Contralateral spinal dorsal horn: GFAP: One-way ANOVA F_3,20_ = 0.5, *P* = 0.686; GFAP positive area: One-way ANOVA F_3,20_ = 49, *P* = 0.69). BMP10 has been established to be expressed in astrocytes (Ogawa et al., 2022). Thus, double-labeled immunofluorescence staining showed that the BMP10 was colocalized with GFAP, which revealed that BMP10 was expressed in astrocytes. In conformity with the western blot results, immunofluorescence staining showed the expression level of BMP10 in the ipsilateral spinal dorsal horn was increased in the SNI mice on postoperative Day 14 compared with sham mice (Figures 3E, F) (BMP10 positive area: Independent two-sample t-test, t = 4.54, *P* = 0.001). Compared with sham mice, the expression level of BMP10 in astrocytes in the ipsilateral spinal dorsal horn of SNI mice was extremely increased (Figure 3G) (colocalized area: Independent two-sample t-test, t = 3.25, *P* = 0.01).

**FIGURE 4:**
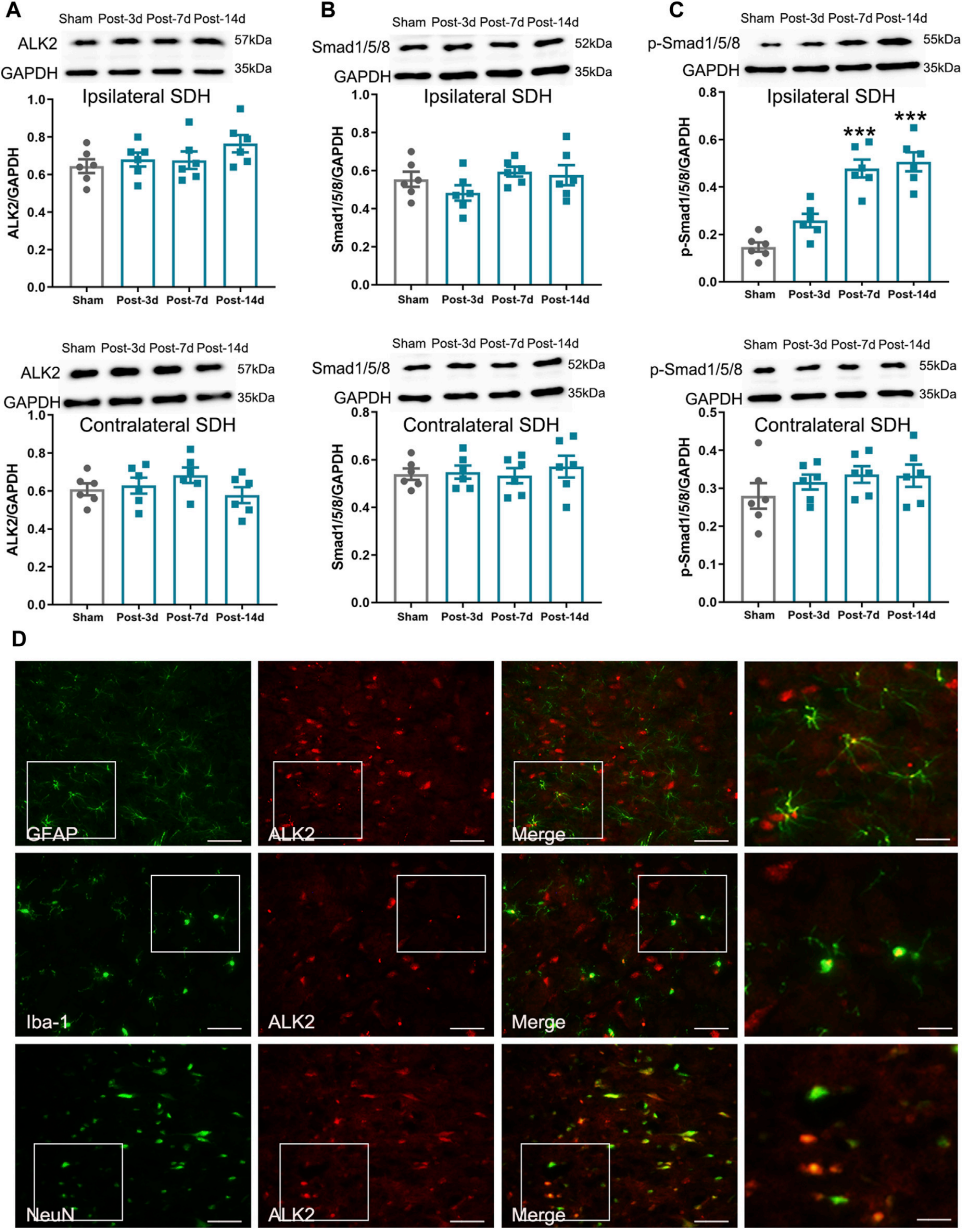
Expression levels of ALK2, Samd1/5/8, and p-Smad1/5/8 in mouse ipsilateral spinal dorsal horn after SNI. **(A–C)** Western blot analysis showed the expression levels of ALK2, Samd1/5/8, and p-Smad1/5/8 in the ipsilateral (above) and contralateral (below) spinal dorsal horn of sham and SNI mice; **(D)** Double immunofluorescence staining showed the coexpression of ALK2 (red) with GFAP, Iba-1 and NeuN (green) in the ipsilateral spinal dorsal horn of SNI mice on postoperative Day 14 (scale bar = 50 μm/25 μm). Data are presented as mean and SEM; sham mice versus SNI mice on postoperative Days 7 and 14, ***p < 0.001 (n = 6).

It is well established that BMP10 affects cell activities by activating intracellular Smad1/5/8 signaling through its specific receptors like ALK1 and ALK2 (Mitrofan et al., 2017; Capasso et al., 2020). As is reported, ALK1 was not expressed in astrocytes, while ALK2 was considered the most widely expressed ALK receptor (Chen et al., 2004; Hamby et al., 2010). The results of double-labeling immunofluorescence showed that ALK2 was colocalized with GFAP, Iba-1(microglial marker), and NeuN (neuronal marker), indicating that ALK2 is expressed on astrocytes, microglia, and neurons in the spinal dorsal horn (Figure 4D). Next, we investigated the activity of ALK2/Smad1/5/8 signaling by detecting the expression of ALK2, Smad1/5/8, and p-Smad1/5/8. In the ipsilateral spinal dorsal horn, the expression of p-Smad1/5/8 was remarkably increased on postoperative Days 7 and 14 (Figure 4C) (Ipsilateral spinal dorsal horn: One-way ANOVA F_3,20_ = 28.84, *P* < 0.0001), while the expressions of ALK2 and Smad1/5/8 remained unchanged after SNI (Figures 4A, B) (Ipsilateral spinal dorsal horn: ALK2: One-way ANOVA F_3,20_ = 1.58, *P* = 0.23; Smad1/5/8: One-way ANOVA F_3,20_ = 1.44, *P* = 0.26). However, the expressions of ALK2, Smad1/5/8, and p-Smad1/5/8 in the contralateral spinal dorsal horn remained unchanged after SNI (Contralateral spinal dorsal horn: ALK2: One-way ANOVA F_3,20_ = 1.27, *P* = 0.312; Smad1/5/8: One-way ANOVA F_3,20_ = 0.25, *P* = 0.860; p-Smad1/5/8: One-way ANOVA F_3,20_ = 0.94, *P* = 0.441). These results indicated that ALK2/Smad1/5/8 signaling activity in the ipsilateral spinal dorsal horn was enhanced after SNI. Moreover, it was possible that BMP10, not ALK2 or Smad1/5/8, contributed to the phosphorylation of Smad1/5/8 in mice after SNI. The similar expressional trend of BMP10, p-Smad1/5/8, and GFAP indicated that BMP10/ALK2/Smad1/5/8 signaling may play a role in astrocytic activation in the ipsilateral spinal dorsal horn of SNI mice.

### 3.3 BMP10 was involved in the pain hypersensitivity and astrocytic activation of SNI mice

**FIGURE 5:**
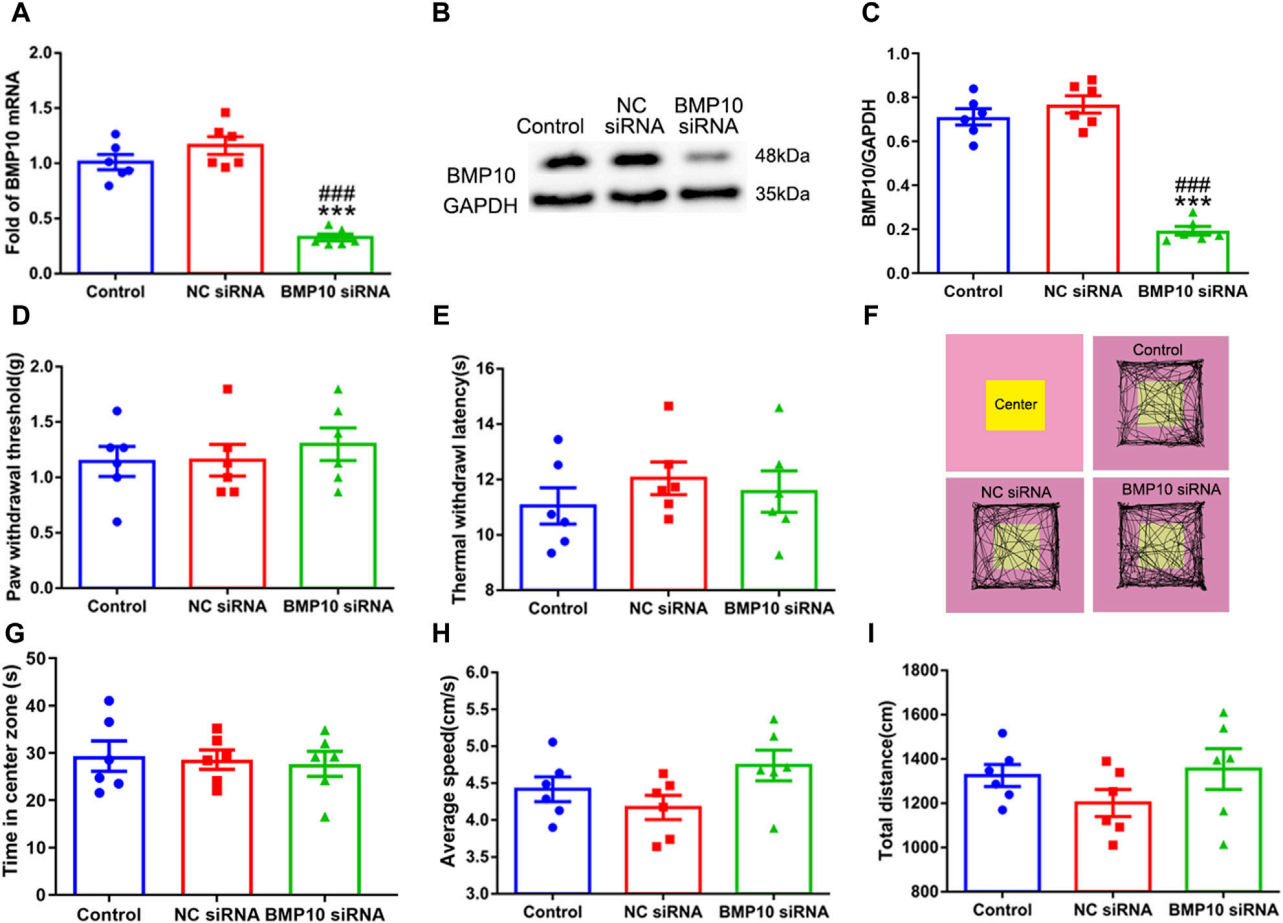
Intrathecal injection of BMP10 siRNA decreased the expression of BMP10 without conspicuous side effects. **(A)** qRT-PCR analysis showed the expression levels of BMP10 mRNA in the spinal cord of mice after intrathecal injection with the vehicle, NC siRNA, and BMP10 siRNA; **(B, C)** Western blot analysis showed the expression levels of BMP10 protein in the spinal cord of mice after intrathecal injected with vehicle, NC siRNA, BMP10 siRNA; **(D, E)** Paw withdrawal threshold (PWT) and thermal withdrawal latency (TWL) of mice after intrathecal injected with vehicle, NC siRNA, BMP10 siRNA; **(F)** Representative movement traces in OFT tests of mice after intrathecal injected with vehicle, NC siRNA, BMP10 siRNA **(G–I)** Time in center zone, average speed, and total distance of mice after intrathecal injected with vehicle, NC siRNA, BMP10 siRNA. Data were presented as mean and SEM, BMP10 siRNA group versus Control group **p < 0.01, ***p < 0.001; BMP10 siRNA group versus NC siRNA group ^##^ *p* < 0.01, ^###^ *p* < 0.001, n = 6.

Following the observation that SNI induced a remarkable upregulation of BMP10 in activated astrocytes, the siRNA targeting BMP10 was used to determine whether BMP10 could modulate astrocytic activation and pain hypersensitivity in SNI mice. First, western blot and qRT-PCR data showed that intrathecal injections of BMP10 siRNA for two consecutive days (4 μg once a day) decreased the BMP10 mRNA and protein expression level in the spinal cord of mice, indicating the successful transfection of BMP10 siRNA (Figures 5A–C) (BMP10 mRNA: One-way ANOVA F_2,15_ = 48.29, *P* < 0.0001; BMP10 protein: One-way ANOVA F_2,15_ = 12.96, *P* = 0.001). In behavioral tests, PWT, TWL, average speed, and total distance of mice intrathecally injected with NC siRNA and BMP10 siRNA were not changed compared with those values of mice in the control group, indicating that NC siRNA or BMP10 siRNA do not have conspicuous side effects on the spinal cord and the adjacent nerves (Figures 5D–F, H, I) (PWT: One-way ANOVA F_2,15_ = 0.37, *P* = 0.699; TWL: One-way ANOVA F_2,15_ = 0.56, *P* = 0.586; Average speed: One-way ANOVA F_2,15_ = 2.51, *P* = 0.115; Total distance: One-way ANOVA F_2,15_ = 1.35, *P* = 0.29). In addition to testing motor function, the OFT is also a classic measurement of anxiety by calculating the time in center zone. Accordingly, there was no significant difference in the time in center zone among the three groups, indicating that knock-down of the BMP10 at the spinal level could not induce anxiety-like behavior (Figure 5G) (Time in center zone: One-way ANOVA F_2,15_ = 0.097 *P* = 0.908).

**FIGURE 6:**
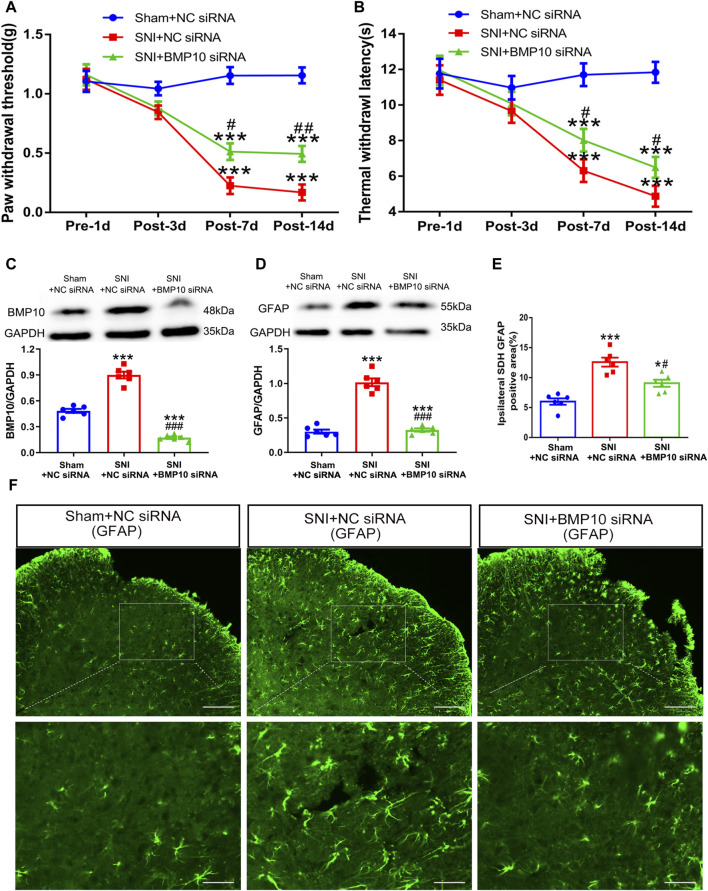
Intrathecal injection of BMP10 siRNA inhibited the SNI-induced pain hypersensitivity and astrocytic activation. **(A, B)** The time course of PWT and TWL in sham and SNI mice after intrathecal delivery of siRNAs; Data are presented as mean and SEM; SNI + NC siRNA group and SNI + BMP10 siRNA group versus Sham + NC siRNA ***p < 0.001; SNI + BMP10 siRNA versus SNI + NC siRNA group ^#^ *p* < 0.05 ^##^ *p* < 0.01 (n = 12); **(C, D)** Western blot showed the expression levels of BMP10 and GFAP in the spinal cord of sham and SNI mice after intrathecal delivery of siRNAs on postoperative Day 14; **(E, F)** Immunofluorescence staining results showing the expression of GFAP in the spinal dorsal horn of sham and SNI mice after intrathecal delivery of siRNAs on postoperative Day 14 (scale bar = 100 μm/50 μm); Data are presented as mean and SEM; SNI + NC siRNA group and SNI + BMP10 siRNA group versus Sham + NC siRNA***p < 0.001; SNI + BMP10 siRNA versus SNI + NC siRNA group ^#^ *p* < 0.05, ^###^ *p* < 0.001 (n = 6).

On postoperative Day 1 and Day 2, 4 μg of NC siRNA or BMP10 siRNA were intrathecally administrated to determine the role of BMP10 in SNI-induced neuropathic pain. On postoperative Day 14, the western blot results showed that the BMP10 expression was dramatically decreased in the spinal cord after intrathecal injection of BMP10 siRNA (Figure 6C) (One-way ANOVA F_2,15_ = 181.55, *P* < 0.0001). As expected, BMP10 siRNA increased the ipsilateral PWT and TWL of SNI mice on postoperative Day 7 and 14 (Figures 6A, B) (PWT: Repeated measure ANOVA F_2,33_ = 34.29, *P* < 0.0001; TWL: Repeated measure ANOVA F_2,33_ = 34.05, *P* < 0.0001). Moreover, intrathecal BMP10 siRNA explicitly inhibited the activation of astrocytes in the ipsilateral spinal dorsal horn of SNI mice on postoperative Day 14 (Figures 6D–F) (GFAP: One-way ANOVA F_2,15_ = 107.81, *P* < 0.0001; GFAP positive area: One-way ANOVA F_2,15_ = 27.13, *P* < 0.0001). These data indicated that BMP10 affected the pain hypersensitivity and astrocytic activation in the spinal dorsal horn of SNI mice.

### 3.4 Blockade of ALK2 reversed BMP10-mediated nociception in normal mice

**FIGURE 7:**
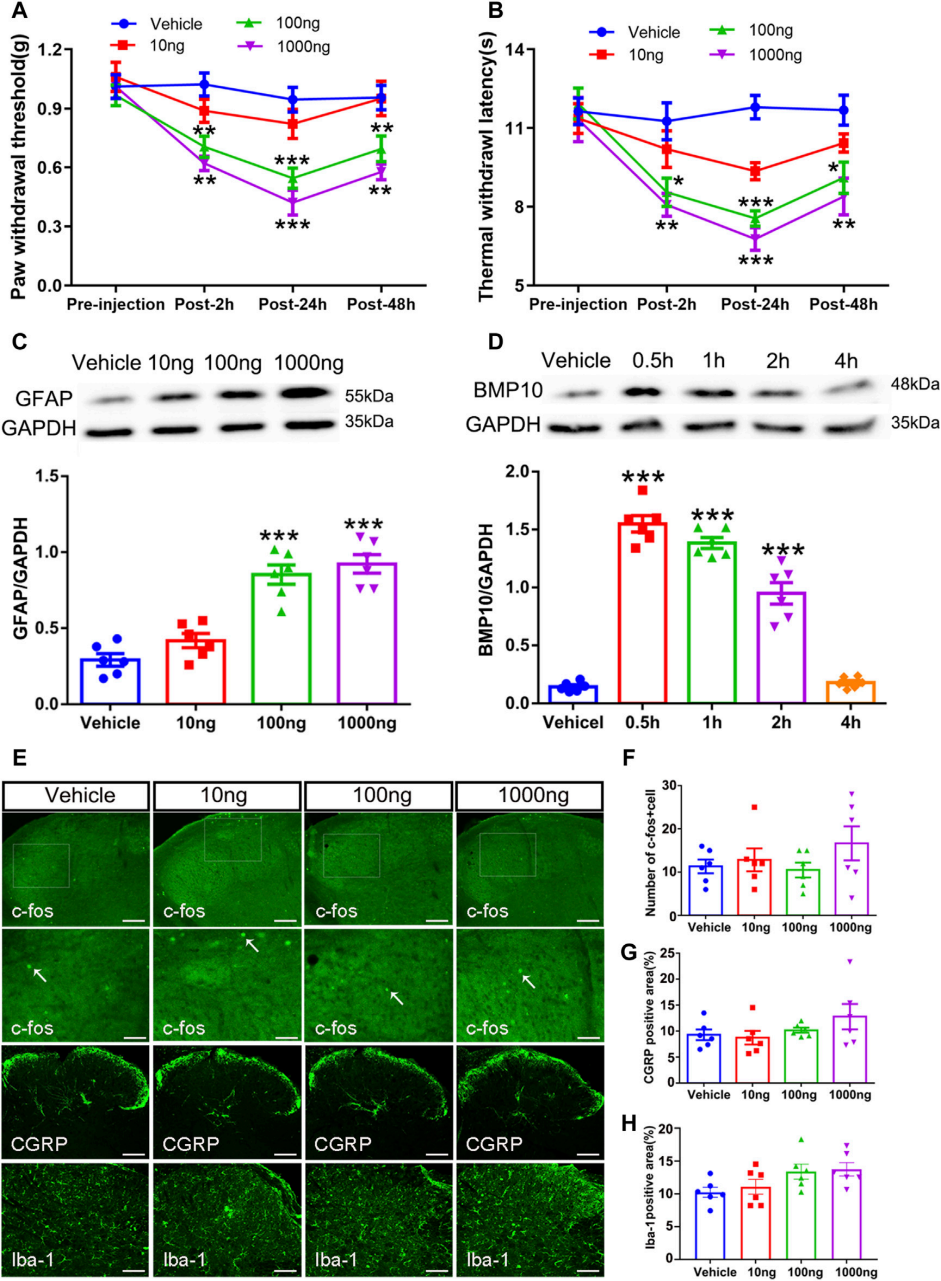
Intrathecal injection of BMP peptide evoked pain hypersensitivity and astrocytic activation in normal mice **(A, B)** The time course of PWT and TWL in normal mice after intrathecal delivery of BMP10 peptide and vehicle; Data were presented as mean and SEM, 100 ng group and 1000 ng group versus Vehicle group *p < 0.05, **p < 0.01, ***p < 0.001; n = 6; **(C)** Western blot showed the expression levels of GFAP in spinal cord of mice after intrathecal delivery of BMP10 peptide and vehicle; **(D)** Western blot showed the expression levels of BMP10 in spinal cord at different time points after intrathecal delivery of BMP10 peptide (100 ng); **(E–H)** Immunofluorescence staining results showing the expression of c-fos, CGRP, and Iba-1 in the spinal dorsal horn of mice after intrathecal delivery of BMP10 peptide and vehicle (scale bar = 200 μm/50 μm). Data were presented as mean and SEM, 100 ng group and 1,000 ng group versus Vehicle group **p < 0.01, ***p < 0.001; n = 6.

Following the investigation that intrathecal injection of BMP10 siRNA could abrogate pain hypersensitivity and astrocytic activation after SNI, we further explored whether a single dose of BMP10 peptide could trigger these effects in normal mice. Normal mice were intrathecally administrated with BMP10 peptide (10 ng, 100 ng, 1,000 ng/2 μL) or vehicle (2 μL of normal saline). The PWT and TWL of bilateral hind paws were then measured at baseline (pre-injection), 2, 24, and 48 h after injection. As shown in Figures 7A, B, changes in PWT and TWL in mice administrated with 10 ng of BMP10 peptide were not significant compared with the vehicle group. However, 100 and 1,000 ng of BMP10 significantly decreased PWT and TWL in normal mice, with the highest decrease observed at 24 h and lasting for at least 48 h compared with the Vehicle group (PWT: Repeated measure ANOVA F_3,44_ = 31.854 *P* < 0.0001; TWL: Repeated measure ANOVA F_3,44_ = 17.15, *P* < 0.0001). Moreover, the marked upregulation of GFAP expression indicated that a single injection of BMP10 peptide (100 ng, 1000 ng) could induce astrocytic activation (Figure 7C) (One-way ANOVA F_3,20_ = 33.99, *P* < 0.0001). Next, we examined the influence of BMP10 on the activity of neurons and microglia in the spinal dorsal horn by detecting the expression of c-fos (a marker of neuron activation), CGRP (nociceptive neurotransmitter), and Iba-1 (microglial marker). However, the results of immunofluorescence staining showed that intrathecal injection of 10, 100, 1,000 ng of BMP10 peptide did not affect the expression of c-fos, CGRP and Iba-1 in spinal dorsal horn (Figures 7E–H) (number of c-fos positive cells: One-way ANOVA F_3,20_ = 1.07, *P* = 0.384; CGRP positive area: One-way ANOVA F_3,20_ = 1.43, *P* = 0.263; Iba-1 positive area: One-way ANOVA F_3,20_ = 2.798, *P* = 0.067). These results indicated that astrocytic activation might be involved in BMP10-induced pain hypersensitivity of normal mice. The bioactivity of exogenetic BMP10 peptide *in vivo* was measured by western blot as previously reported (Zhang et al., 2022). The BMP10 protein level in the spinal cord was remarkably increased at 0.5, 1, and 2 h after BMP10 peptide (100 ng) injection compared with the vehicle group (Figure 7D) (One-way ANOVA F_4,25_ = 132.45, *P* < 0.0001). This result implied that the bioactivity of exogenetic BMP10 peptide in the spinal cord could be maintained for approximately 2 h.

**FIGURE 8:**
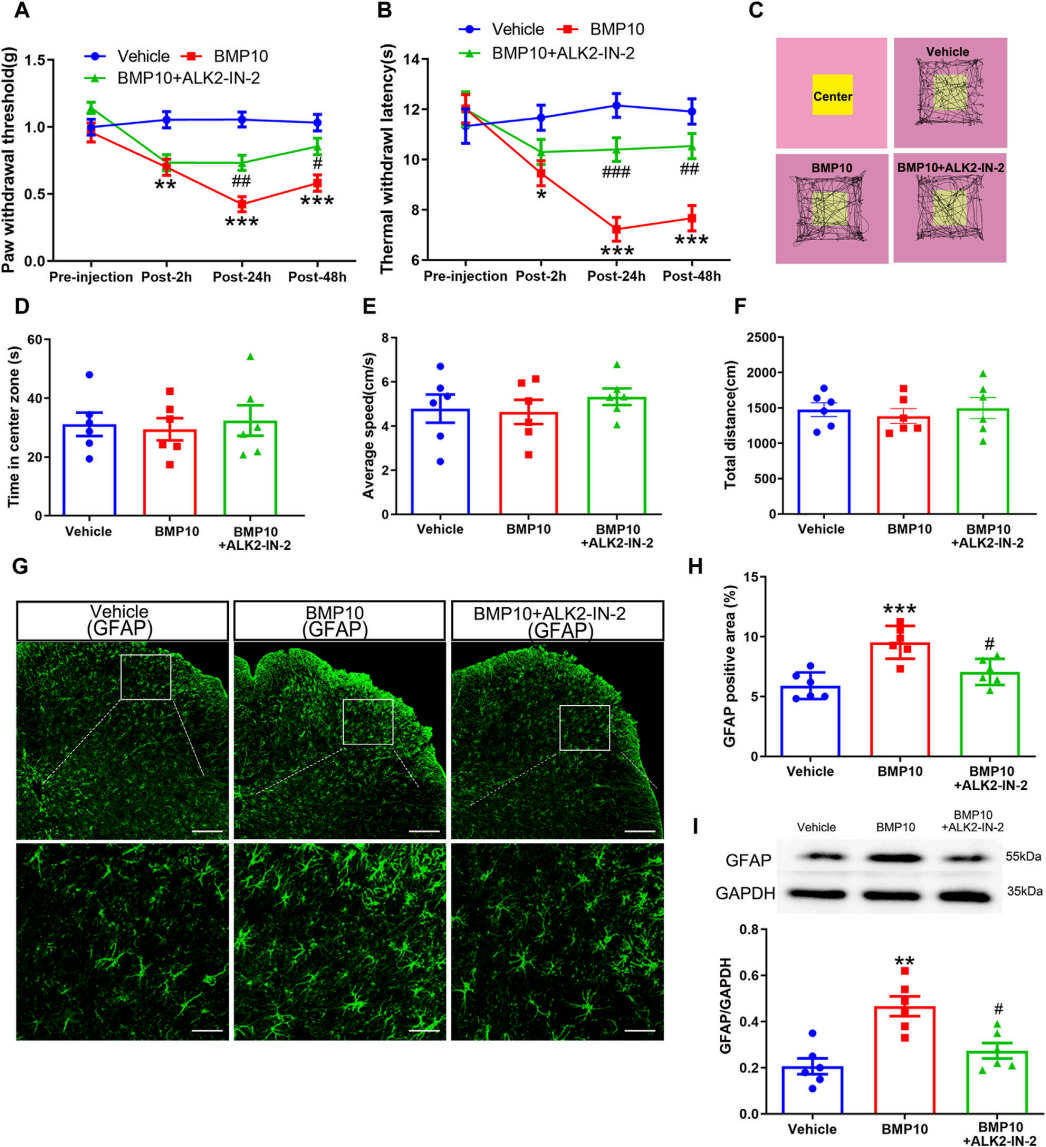
ALK2 was involved in BMP10-induced pain hypersensitivity and astrocytic activation. **(A, B)**The time course of PWT and TWL in normal mice after intrathecal delivery of BMP10 peptide and reagents; Data are presented as mean and SEM, Vehicle group versus BMP10 group *p < 0.05, **p < 0.01, ***p < 0.001; BMP10+ALK2-IN-2 group versus BMP group ^#^ *p* < 0.05,^##^ *p* < 0.01,^###^ *p* < 0.001 (n = 12); **(C)** Representative movement traces in OFT tests of mice after intrathecal delivery of BMP10 peptide and reagents; **(D–F)** Time in center zone, average speed and total distance of mice in open field after intrathecal delivery of BMP10 peptide and reagents; **(G, H)** immunofluorescence staining showed the expression of GFAP in the spinal dorsal horn after intrathecal delivery of BMP10 peptide and reagents (scale bar = 200/50 μm); **(I)** Western blot showed the expression of GFAP in the spinal cord after intrathecal delivery of BMP10 peptide and reagents; Data are presented as mean and SEM, Vehicle group versus BMP10 group *p < 0.05, **p < 0.01, ***p < 0.001; BMP10+ ALK2-IN-2 group versus BMP group ^#^ *p* < 0.05,^##^ *p* < 0.01,^###^ *p* < 0.001 (n = 6).

To investigate the involvement of ALK2 in BMP10-mediated astrocytic activation, the BMP10 (100 ng) treated normal mice were co-administrated with 100 ng of high selective ALK2 inhibitor (ALK2-IN-2). As shown in Figure 7, BMP10-induced pain hypersensitivity (Figures 8A, B) and astrocytic activation (Figures 8G–I) were reversed by inhibition of ALK2 (PWT: Repeated measure ANOVA F_2,33_ = 20.92, *P* < 0.0001; TWL: Repeated measure ANOVA F_2,33_ = 23.16, *P* < 0.0001; GFAP: One-way ANOVA F_2,15_ = 13.089, *P* = 0.0001; GFAP positive area: One-way ANOVA F_2,15_ = 14.455, *P* < 0.0001). OFT was performed following the last PWT and TWL tests, the results of OFT showed that intrathecal injection of BMP10 peptide, ALK2-IN-2 did not cause any motor impairment and anxiety-like behavior (Figures 8C–F) (Time in center zone: One-way ANOVA F_2,15_ = 0.49, *P* = 0.622; Average speed: One-way ANOVA F_2,15_ = 0.287, *P* = 0.754; Total distance: One-way ANOVA F_2,15_ = 0.463, *P* = 0.638). Collectively, these data indicated that ALK2 acts as a specific binding receptor, mediating the BMP10’s effects in SNI mice.

### 3.5 Smad1 regulated astrocytic activation *in vitro*

**FIGURE 9:**
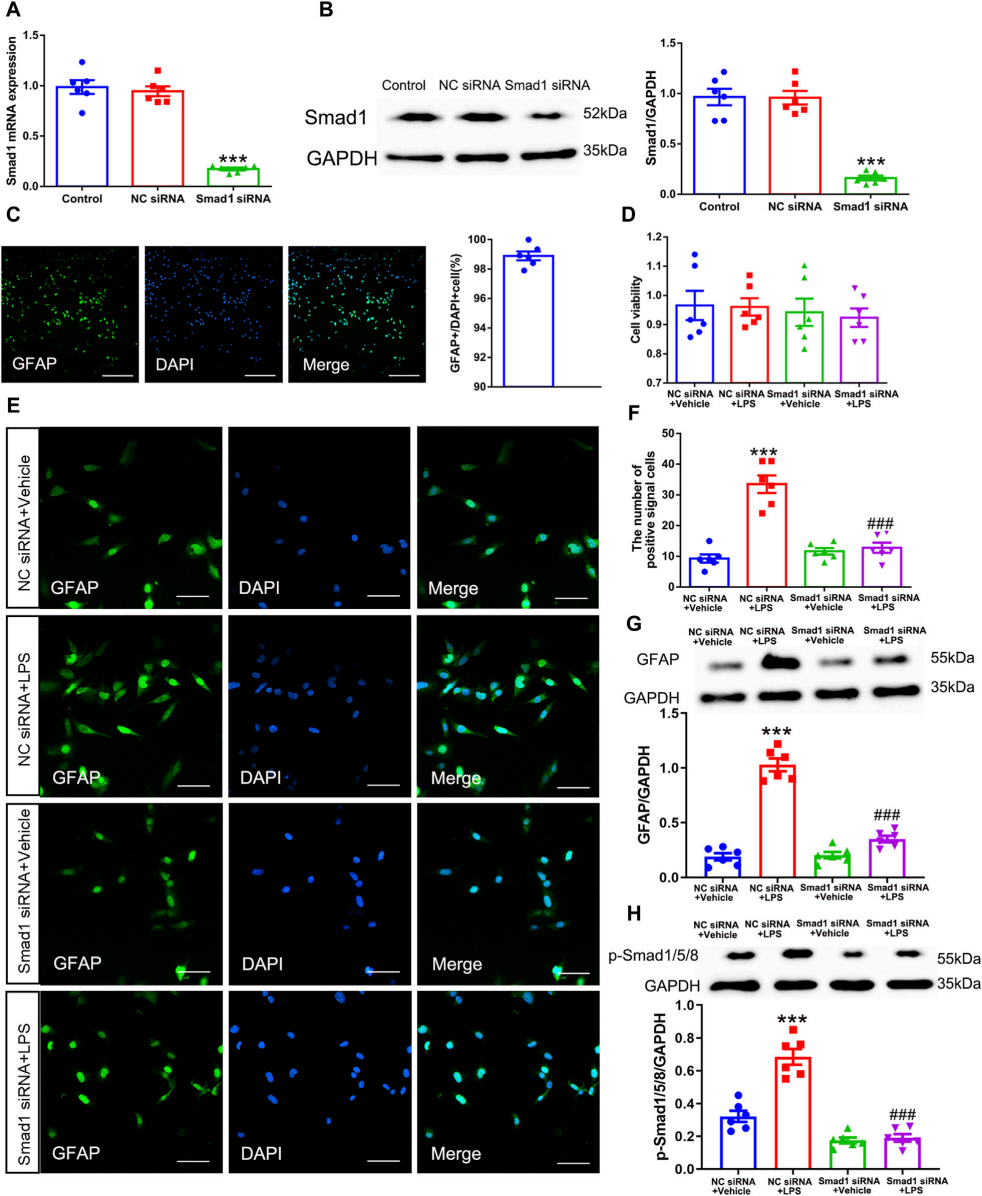
Smad1 was pivotal in astrocytic activation *in vitro*. **(A)** qRT-PCR analysis showed the expression levels of Smad1 mRNA in cultured astrocytes transfected with siRNAs; **(B)** Western blot analysis showed the expression levels of Smad1 protein in cultured astrocytes transfected with siRNAs data are presented as mean and SEM, Smad1 siRNA group versus + Control group ***p < 0.001 (n = 6); **(C)** The purity of cultured astrocytes (scale bar = 200 μm); **(D)** The CCK assay showed the cell viability of cultured astrocytes; **(E, F)** Immunofluorescence staining results showed the expression of GFAP in cultured astrocytes transfected with siRNAs (scale bar = 50 μm); **(G, H)** Western blot showed the expressions of GFAP and p-Smad1/5/8 and in cultured cells; data are presented as mean and SEM, NC siRNA + Vehicle group versus NC siRNA + LPS group ***p < 0.001; NC siRNA + LPS group versus Smad1 siRNA + LPS group ^###^ *p* < 0.001 (n = 6).

Having confirmed that phosphorylation of Smad1/5/8 was enhanced after SNI, the role of Smad1/5/8 in astrocytic activation was further investigated *in vitro* by transfecting with Smad1 siRNA. Accordingly, Smad1 siRNA and NC siRNA were transfected into cultured mouse spinal cord astrocyte cell line (purity>97%, 1 × 10^5^/ml, Figure 9C). The western blot and qRT-PCR results showed that Smad1 siRNA successfully knocked down the expression of Smad1 in cultured cells without obvious influence on cell viability (Figures 9A, B, D) (Smad1 mRNA: one-way ANOVA F_2,15_ = 87.382, *P* < 0.0001; Smad1 protein: one-way ANOVA F_2,15_ = 53.997, *P* < 0.0001; cell viability: one-way ANOVA F_2,15_ = 0.080, *P* = 0.923). As described previously, LPS was a potent stimulator for cultured astrocytes (Xian et al., 2019). Therefore, we cultured the Smad1 siRNA- and NC siRNA-transfected astrocytes with 100 ng/mL LPS to examine the role of Smad1 in astrocytic activation. Notably, LPS remarkably promoted the expression of GFAP and p-Smad1/5/8 in NC siRNA-transfected astrocytes (Figures 9E–H), indicating the stimulative effects of LPS (100 ng/mL) on activation of cultured astrocytes and phosphorylation of Smad1/5/8. However, LPS-induced *in vitro* activation of astrocytes and phosphorylation of Smad1/5/8 was blocked by transfection with Smad1 siRNA (Figures 9E–H) (Cell number: one-way ANOVA F_3,20_ = 36.144, *P* < 0.0001; p-Smad1/5/8: one-way ANOVA F_3,20_ = 51.626, *P* < 0.0001; GFAP: one-way ANOVA F_3,20_ = 101.62, *P* < 0.0001). These results indicated that Smad1/5/8 was pivotal in the activation of astrocytes.

### 3.6 Knocking down Smad1 suppressed BMP10-induced activation of astrocytes

**FIGURE 10:**
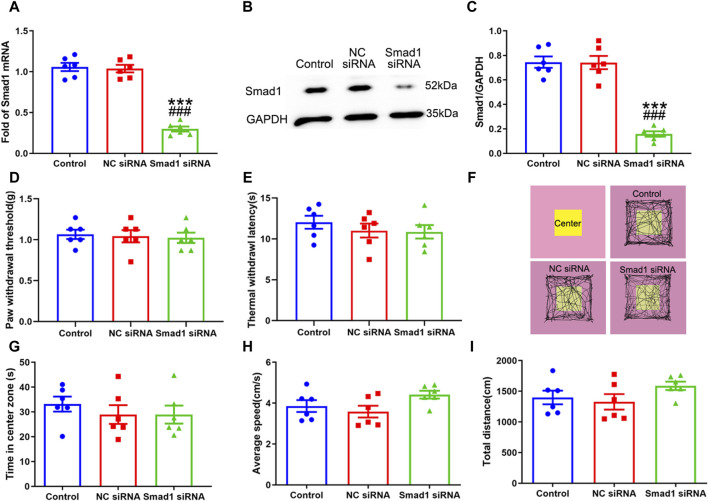
Intrathecal injection of Smad1 siRNA knocked down the expression of Smad1 without obvious side effects. **(A)** qRT-PCR analysis showed the expression levels of Smad1 mRNA in the spinal cord of mice after intrathecal injection with vehicle, NC siRNA, Smad1 siRNA; **(B, C)** Western blot analysis showed the expression levels of Smad1 protein in the spinal cord of mice after intrathecal injected with vehicle, NC siRNA, Smad1 siRNA; **(D, E)** Paw withdrawal threshold (PWT) and Thermal withdrawal latency (TWL) of mice after intrathecal injected with vehicle, NC siRNA, Smad1 siRNA **(F)** Representative movement traces in OFT tests of mice after intrathecal injected with vehicle, NC siRNA, Smad1 siRNA **(G–I)** Time in center zone, average speed and total distance of mice after intrathecal injected with vehicle, NC siRNA, Smad1 siRNA. Data were presented as mean and SEM, Smad1 siRNA group versus Control group ***p < 0.001; Smad1 siRNA group versus NC siRNA group ^###^ *p* < 0.001, n = 6.

**FIGURE 11:**
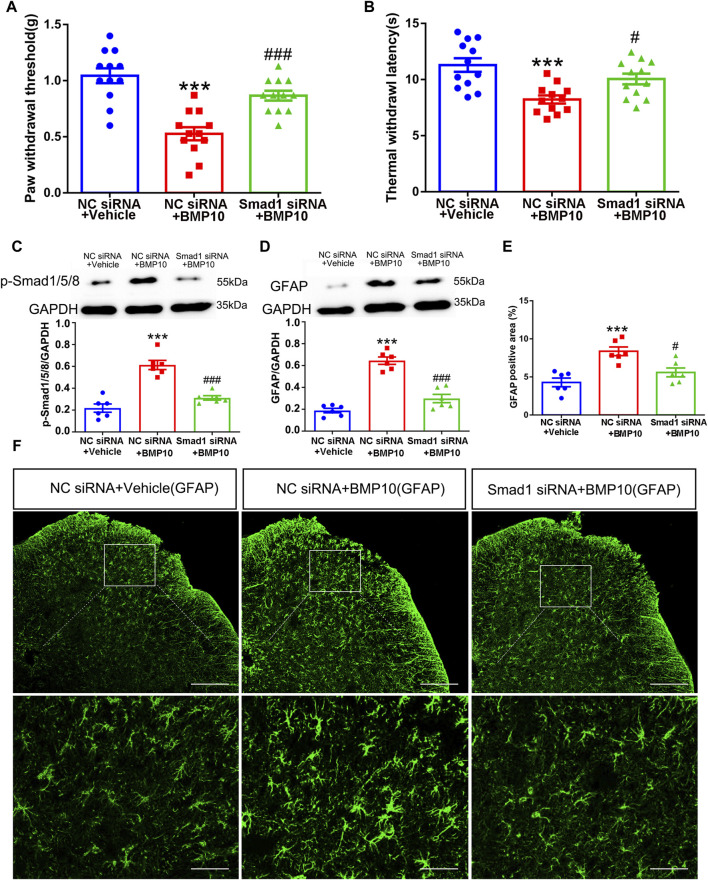
Smad1 was involved in BMP10-induced pain hypersensitivity and astrocytic activation. **(A, B)** PWT and TWL of normal mice after intrathecal delivery of siRNA and BMP10 peptide; **(C, D)** Western blot showed the expressions of p-Smad1/5/8 and GFAP in the spinal cord of normal mice after intrathecal delivery of siRNA and BMP10 peptide; **(E, F)** immunofluorescence staining showed the expression of GFAP in the spinal dorsal horn of normal mice after the intrathecal delivery of siRNA and BMP10 peptide; (scale bar = 200 μm/50 μm); Data are presented as mean and SEM, NC siRNA + Vehicle group versus NC siRNA + BMP10 group ***p < 0.001; NC siRNA + BMP10 group versus Smad1 siRNA + BMP10 group ^#^ *p* < 0.05,^###^ *p* < 0.001 (n = 6).

Having confirmed that Smad1/5/8 plays a key role in LPS-induced astrocytic activation *in vitro* by transfection with Smad1 siRNA, Smad1 siRNA was intrathecally administered to examine whether Smad1 was involved in BMP10-induced activation of spinal astrocytes *in vivo*. Smad1 siRNA (4 μg once a day) was intrathecally administrated for two consecutive days to knock down the expression of Smad1 in the spinal cord before BMP10 administration. Notably, the spinal expression of Smad1 mRNA and protein were decreased on 2 days after Smad1 siRNA transfection (Figures 10A–C) (Smad1 mRNA: one-way ANOVA F_2,15_ = 99.993, *P* < 0.0001; Smad1 protein: one-way ANOVA F_2,15_ = 61.29, *P* < 0.0001). In behavioral tests, bilateral PWT and TWL, average speed, and total distance of mice intrathecally injected with NC siRNA and Smad1 siRNA were not changed compared with those values of mice in the control group, indicating that NC siRNA or Smad1 siRNA do not have obvious motor and sensory side effects (Figures 10D–F, H, I) (PWT: One-way ANOVA F_2,15_ = 0.110, *P* = 0.897; TWL: One-way ANOVA F_2,15_ = 0.587, *P* = 0.569; Average speed: One-way ANOVA F_2,15_ = 2.633, *P* = 0.105; Total distance: One-way ANOVA F_2,15_ = 1.653, *P* = 0.225). Moreover, there was no significant difference in the time in center zone among the three groups, indicating that knock-down of the Smad1 at the spinal level could not induce anxiety-like behavior (Figure 10G) (Time in center zone: One-way ANOVA F_2,15_ = 0.117, *P* = 0.892). To investigate the role of Smad1/5/8 in BMP10’s effects, 100 ng of BMP10 peptide was intrathecally administrated after intrathecal injection of Smad1 siRNA or NC siRNA for two consecutive days. The behavioral tests were performed at 24 h after BMP10 peptide administration. Notably, the BMP10 peptide reduced the threshold toward mechanical and thermal stimuli in mouse paws (Figures 11A, B) and promoted the activation of astrocytes in the spinal dorsal horn (Figures 11D–F) (PWT: One-way ANOVA F_2,33_ = 20.510, *P* < 0.0001; TWL: One-way ANOVA F_2,33_ = 9.892, *P* = 0.001; GFAP: One-way ANOVA F_2,15_ = 56.73, *P* < 0.0001; GFAP positive area: One-way ANOVA F_2,15_ = 13.160, *P* = 0.001). In addition, BMP10 also facilitated the phosphorylation of Smad1/5/8 (Figure 11C) (p-Smad1/5/8: One-way ANOVA F_2,15_ = 30.244, *P* < 0.0001). However, BMP10-induced hyperalgesia, astrocytic activation, and phosphorylation of Smad1/5/8 did not happen in mice receiving Smad1 siRNA transfection (Figure 11). These data indicated that Smad1/5/8 is a key modulator of BMP10 induced-astrocytic activation.

## 4 Discussion

Increasing evidence has demonstrated that BMPs are involved in neuropathic pain. However, the potential mechanisms are multifarious and complex. In the present study, we first confirmed that BMP10 was obviously upregulated in activated astrocytes of mice’s ipsilateral spinal dorsal horn after SNI. Knocking down spinal BMP10 alleviated pain hypersensitivity after SNI, probably by restraining the activation of astrocytes. In addition, the BMP10 peptide exhibited the utility to evoke pain hypersensitivity and astrocytic activation through its ALK2 binding receptor. Furthermore, targeting Smad1 *in vitro* using siRNA potently reversed the activation of astrocytes induced by LPS. Finally, knocking down Smad1 by suppressing astrocytic activity vividly abrogated the BMP10-induced hypersensitivity. Taken together, these results suggest that the BMP10/ALK2/Smad1 axis plays a key role in pain hypersensitivity after peripheral nerve injury, which points to its stimulative ability to astrocyte.

BMPs, the important regulators of bone formation, are increasingly recognized to be involved in multiple neurological processes (Nakashima et al., 2021; Wang et al., 2022). With regard to neuropathic pain, some BMP subtypes are reported to exhibit different effects on pain sensitization after nerve injury. For example, BMP7 has been demonstrated to be a protective regulator against neuropathic pain caused by sciatic nerve injury in mice, partly owing to its facilitation of endogenous opioid-dependent analgesic system (de la Puerta et al., 2019). In contrast, intrathecal injection of exogenous BMP4 elicited tactile allodynia and activation of spinal astrocytes by promoting Smad1/5/8 phosphorylation (Yang et al., 2019). So far, BMP7 and BMP4 are the only BMP family members whose vital regulatory roles in neuropathic pain have been uncovered in some depth. Herein, we found that BMP10 is gradually upregulated in the ipsilateral spinal dorsal horn of mice with SNI. This increasing trend pointed to the possible involvement of BMP10 in neuropathic pain. Although little information is available about the physiological role of BMP10 in the CNS, a recent study revealed the abundant expression of BMP10 in the brain and spinal cord of adult rats (Ogawa et al., 2022). However, BMP10 had not been found in the CNS of the mouse embryo (Neuhaus et al., 1999). Therefore, the expressional profile indicates that BMP10 might modulate the function of CNS in adults. Interestingly, BMP10 was also found to be upregulated in rats subjected to traumatic brain injury and contributed to its astrocytic activation in the lesion site (Yan et al., 2012). In this study, the dramatically upregulated expression of GFAP indicated the astrocytic activation in the ipsilateral spinal dorsal horn after SNI. In addition, our double immunostaining data revealed that the upregulation of BMP10 was consistent with the activation of astrocytes, as shown by its colocation with astrocytes. It is well known that the activated astrocytes contribute to the central sensitization of neuropathic pain by releasing pain mediators such as proinflammatory cytokines in the spinal dorsal horn (Cheng et al., 2022). Therefore, the expressional profile of BMP10 in the spinal dorsal horn of mice with SNI points to the possibility of BMP10 being relevant to the activation of astrocytes after SNI. As expected, the intrathecal administration of BMP10 siRNA ameliorated SNI-induced pain hypersensitivity and astrocytic activation in the ipsilateral spinal dorsal horn. Moreover, intrathecal injection of exogenous BMP10 peptide decreased the sensory threshold toward paw mechanical and thermal stimuli and promoted the activation of astrocytes in the spinal dorsal horns of normal mice. Therefore, these results demonstrated the involvement of BMP10 in SNI-induced neuropathic pain and astrocytic activation. It should be noted that the overexcitability of sensory neurons and aberrant activation of microglia also contribute to pain sensitization in the spinal dorsal horn (Finnerup et al., 2021). In the current study, BMP10 could not affect the activity of neurons and microglia at the spinal level by the observation of the unchanged CGRP (a neurotransmitter secreted from the hyperexcited Aδ and C fibers) (Iyengar et al., 2017), c-fos (a marker of neuronal activation) (Wang et al., 2023), and Iba-1 (microglial marker). Collectively, these results suggest that BMP10 is a mediator that modulates neuropathic pain, possibly through the activation of astrocytes.

The action of BMP10 is required for the intracellular downstream signaling mediators, such as ALK binding receptors and Smad proteins (Li et al., 2016; Capasso et al., 2020). It is well established that ALK1 and ALK2 are the most reported TGF-β type-1 receptors that initiate BMP10-induced phosphorylation of Smad1/5/8 (Mitrofan et al., 2017; Capasso et al., 2020). However, ALK1 was demonstrated not to be expressed in astrocytes (Hamby et al., 2010). In the current study, ALK2 was shown to be widely expressed on astrocytes, microglia, and neurons in the spinal dorsal horn and remained unchanged after SNI. As reported previously, ALK2 was involved in the BMPs-mediated astrocytic differentiation (Brederlau et al., 2004). To determine the participation of ALK2 in BMP10-induced astrocytic activation, the BMP10-treated normal mice were co-administrated with ALK2 selective inhibitor (ALK2-IN-2). Here, we found that blocking ALK2 at the spinal cord level could abrogate BMP10-induced astrocytic activation and pain hypersensitivity. So far, these results confirmed that ALK2 was the specific binding receptor for BMP10, which initiated astrocytic activation and pain hypersensitivity. The regulation of BMP10 is mainly performed on the transcription level of astrocytes. Smad1/5/8 phosphorylation is the intracellular signaling pathway directly associated with BMP10-induced transcriptional expression. Consistent with the upregulation of BMP10, we found that the expression levels of phosphorylated Smad1/5/8 in the spinal dorsal horn were time-dependently increased after SNI. We next found that Smad1/5/8 was indispensable for the activation of astrocytes by transfecting the Smad1 siRNA into cultured astrocytes. In line with our results, the role of Smad1/5/8 in astrocytic differentiation and activation is well established (North et al., 2015; Meares et al., 2018). To further examine the fact that Smad1/5/8 also plays a critical role in the BMP10-induced astrocytic activation, Smad1 siRNA was intrathecally administrated to knock down the expression of Smad1 in the spinal cord. As expected, Smad1 siRNA also reversed the BMP10-induced hyperalgesia and astrocytic activation in the spinal dorsal horn of normal mice.

## 5 Conclusion

In summary, this study shed light on the critical role of BMP10 in pain sensitization of SNI mice, and the mechanisms depended on its facilitation of astrocytic activation via ALK2/Smad1/5/8 signaling. These findings provide a potential mechanistic basis for the observed pathological changes in neuropathic pain and highlight the possibility of BMP10 being a therapeutic target in the pharmacological treatment of neuropathic pain.

## 6 Limitations

The upregulated BMP10 in the spinal dorsal horn contributed to the development of neuropathic pain. However, the reason why SNI induced the upregulation of BMP10 is still unknown. In addition, the underlying pathway of BMP10-induced astrocytic activation needs more evidence. Furthermore, we haven't investigated whether other BMP receptors or their relevant intracellular signaling involved BMP10-induced pain hypersensitivity or astrocytic activation. These issues will be explored in our future study.

## Data Availability

The raw data supporting the conclusions of this article will be made available by the authors, without undue reservation.

## Supplementary material

SUPPLEMENTARY FIGURE S1 Full-length blots in Figure 3.

SUPPLEMENTARY FIGURE S2 Full-length blots in Figure 4.

SUPPLEMENTARY FIGURE S3 Full-length blots in Figures 5–8.

SUPPLEMENTARY FIGURE S4 Full-length blots in Figures 9–11.
