# Supplementary figures and images for: BMP10 accelerated spinal astrocytic activation in neuropathic pain via ALK2/smad1/5/8 signaling
Source: Front Pharmacol. 2024 Aug 12;15:1426121. doi: 10.3389/fphar.2024.1426121 (PMC11345179; doi:10.3389/fphar.2024.1426121)

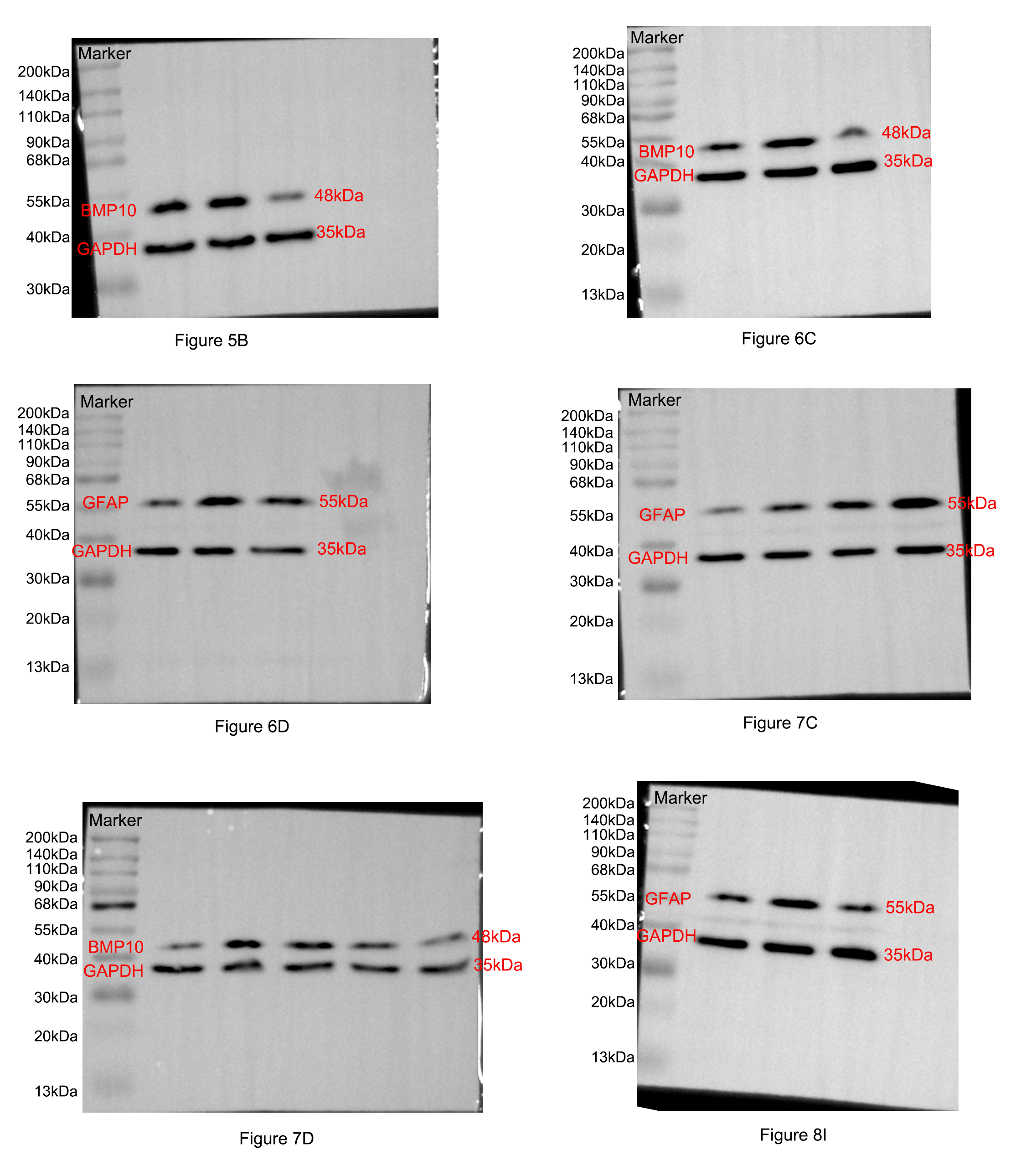

Supplement: Supplementary file 1 [file Image3.TIF]

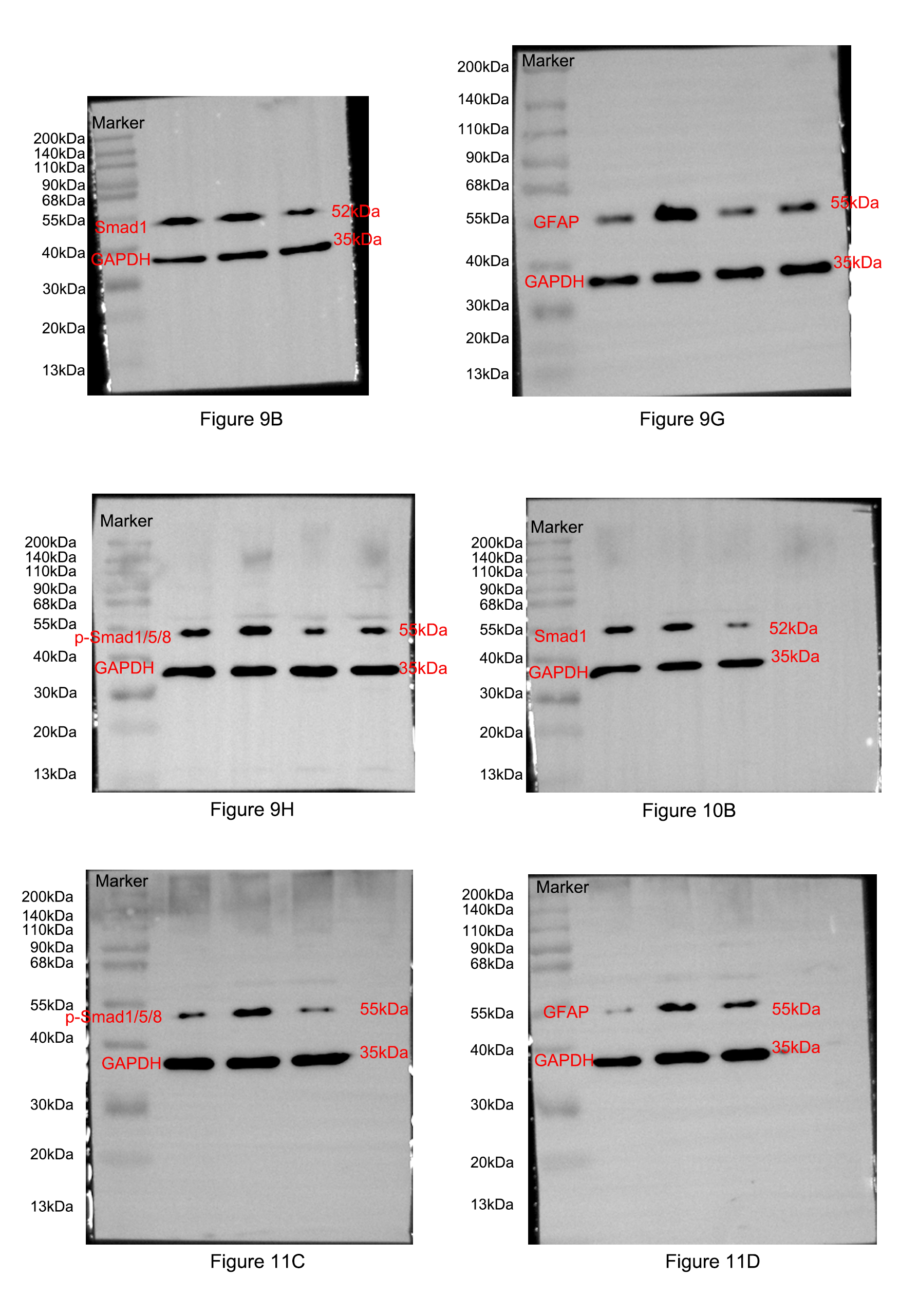

Supplement: Supplementary file 2 [file Image4.TIF]

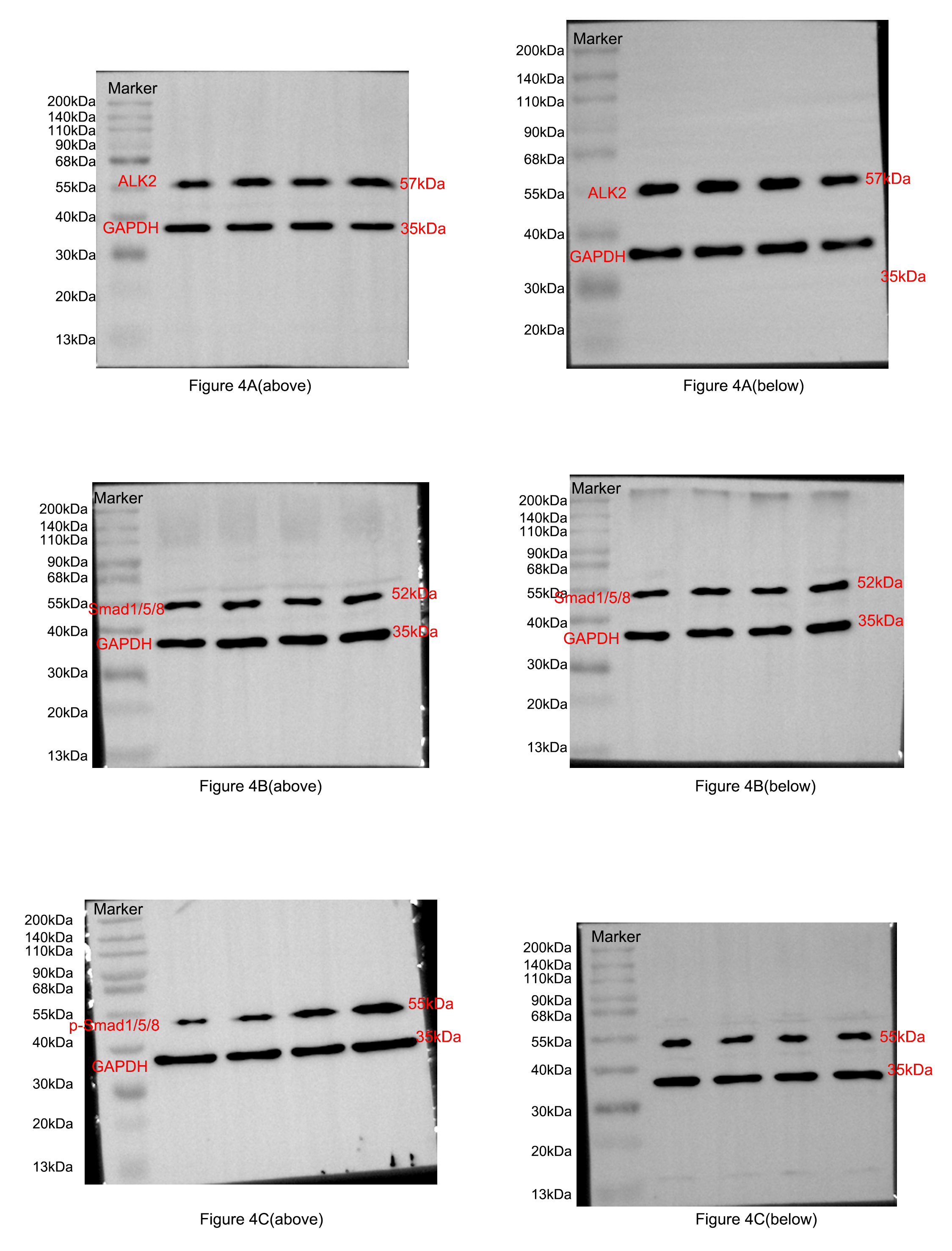

Supplement: Supplementary file 3 [file Image2.TIF]

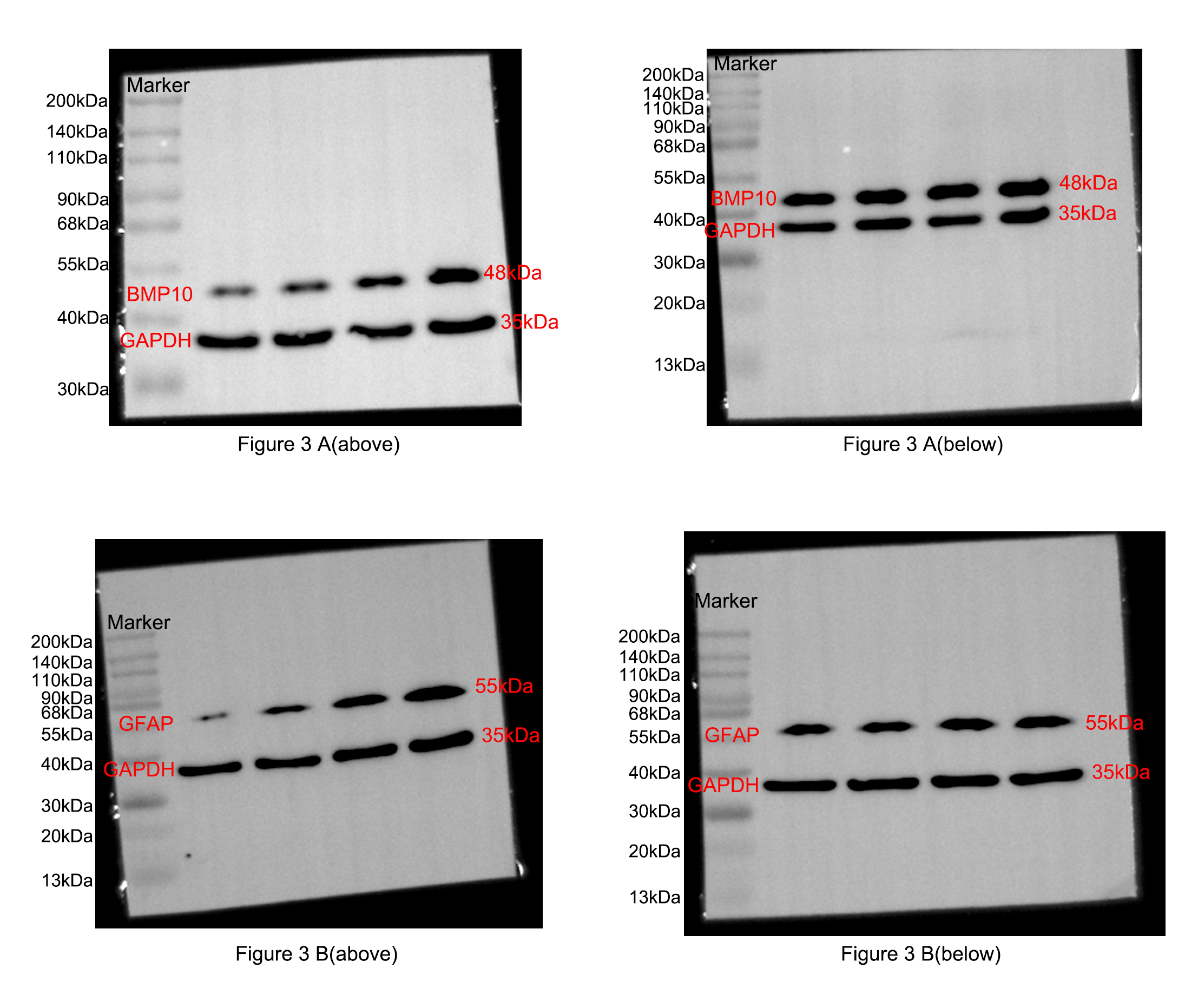

Supplement: Supplementary file 4 [file Image1.TIF]
